# Stoichiometric MACl/PbCl_2_ Additive Engineering for Improved Reproducibility and Carrier Lifetime in FA/Cs Mixed-Halide Perovskite Solar Cells

**DOI:** 10.1021/acsomega.6c01439

**Published:** 2026-07-29

**Authors:** Vishal Viswakarman, Miguel García Rocha, Johann Bouclé

**Affiliations:** † 681581 Centro de Investigación y de Estudios Avanzados del Instituto Politécnico Nacional (Cinvestav), Program on Nanoscience and Nanotechnology, Av. Instituto Politécnico Nacional, 2508, Col. San Pedro Zacatenco, Ciudad de México, Código Postal 07360, México; ‡ Centro de Investigación y de Estudios Avanzados del Instituto Politécnico Nacional (Cinvestav), Department of Physics, Av. Instituto Politécnico Nacional, 2508, Col. San Pedro Zacatenco, Ciudad de México, Código Postal 07360, México; § Univ. Limoges, CNRS, XLIM, UMR 7252, F-87000 Limoges, France

## Abstract

Chloride-based additive engineering has become nearly indispensable in the development of high-efficiency perovskite solar cells (PSCs), with the majority of record-setting devices achieving power conversion efficiencies (PCE) exceeding 25% using methylammonium chloride (MACl) as a processing additive. However, the extreme process sensitivity and consequent irreproducibility associated with MACl incorporation are rarely addressed. Here, we systematically investigate MACl-processed FA_0.9_Cs_0.1_Pb­(I_0.9_Br_0.1_)_3_ films and show that MACl–assisted processing can act as a double-edged strategy. Under nominally identical processing conditions, a minority of samples exhibit strong photoluminescence (PL) enhancement, whereas the majority display PL quenching despite their larger grain sizes. Using complementary techniques such as X-ray diffraction (XRD), line-profile analysis, atomic force microscopy (AFM), and time-resolved PL (TRPL), we attribute this bimodal response to compositional and structural heterogeneity, including halide-segregated domains and nonuniform microstrain that increase trap-assisted nonradiative recombination. To mitigate this instability, we replace volatile MACl with a reduced-volatility chloride additive derived from a stoichiometric MACl/PbCl_2_ precursor (1:1 molar ratio, hereafter MCP chloride additive). At optimal additive concentration (5 mol %), the MCP-5 yields single-phase diffraction signatures, homogenized PL, and substantially extended carrier lifetimes, increasing the average lifetime by a factor of ∼7–9 (from ∼180 ns to ∼1.3–1.6 μs). In n–i–p PSCs, MCP-5-based devices yield modest but representative efficiencies, with a best PCE of 12.03%, a ∼20% relative efficiency gain, and a 30 mV increase in open-circuit voltage compared to MACl-20-treated devices, together with reduced device-to-device variability. Short-term humidity tests on unencapsulated cells (70–75% relative humidity, 24 h) further show improved PCE retention for MCP–5 compared to MACl–20 and control devices. Overall, these results identify MACl-assisted processing-induced heterogeneity as a key source of irreproducibility and establish MCP chloride additive processing routes as a promising approach to more reproducible mixed-halide PSCs, while highlighting the need for future long-term stability studies.

## Graphical abstract

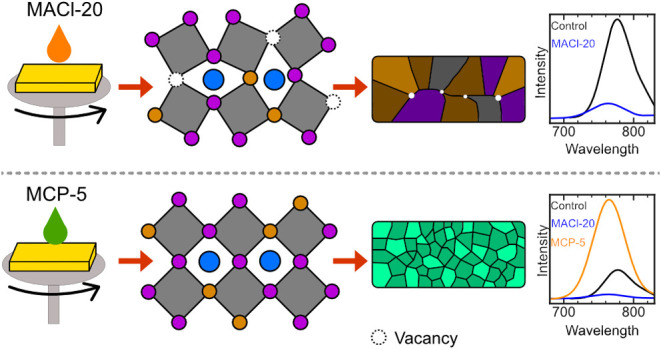

## Introduction

In the rapidly evolving landscape of renewable energy, organic–inorganic hybrid metal halide perovskites (MHPs) with the general formula ABX_3_ have emerged as one of the most promising alternatives to the well-established silicon-based photovoltaic technologies. Beyond solar energy, the ABX_3_ framework is recognized for its immense structural versatility, supporting diverse physical phenomena ranging from optoelectronics to superconductivity.^1^ Perovskite solar cells (PSCs) have witnessed remarkable performance improvements, with certified power conversion efficiencies (PCEs) for single-junction devices now exceeding 27%.^2–4^ While hybrid lead-halide perovskites dominate the field, significant research has turned toward lead-free alternatives to address toxicity and stability concerns.^5–8^ To advance the commercialization of PSCs, it is essential to enhance both their efficiency and long-term stability simultaneously. In recent years, mixed-cation mixed-halide (MCMH) perovskite compositions have garnered significant attention owing to their improved phase stability, enhanced thermal and environmental resilience, tunable band gaps, and versatility for tandem device architectures.^9–11^ Specifically, alloying FA^+^ with smaller A-site cations such as Cs^+^ and MA^+^, and substituting I^–^ with Br^–^ or Cl^–^ at the X-site, enables optimization of the Goldschmidt tolerance factor, stabilization of the photoactive perovskite phase, and precise bandgap engineering.^12–16^ However, due to ionic size mismatches among A-site cations, high bromine (Br) content, and the low solubility and small ionic radius of Br, MCMH perovskites often undergo rapid crystallization and halide redistribution, resulting in energetically disordered films with reduced grain sizes and nonuniform phase distributions.^17–20^ These structural defects serve as nonradiative recombination centers, thereby directly affecting the open-circuit voltage (*V*_OC_) and fill factor (FF) of the resulting devices.^21,22^ Furthermore, compositional inhomogeneity present in as-fabricated films creates an energetically inhomogeneous landscape that accelerates light-induced halide phase segregation during operation, degrading long-term stability.^23–26^ Moreover, the lattice mismatch among the mixed ions induces strain, a double-edged sword that can either enhance phase stability or, if uncontrolled, promote defects, ion migration, and, hence, device degradation.^27–30^ Hence, strategies for defect passivation, crystallization, and strain control are essential for achieving efficient, stable, and defect-free MCMH perovskites.

Various strategies, including additive engineering,^31,32^ defect passivation,^33,34^ and strain engineering,^30,35^ are utilized to decrease defect density, regulate crystallization, and enhance the crystallinity of perovskite films. Notably, additive engineering is recognized as a robust and effective strategy to address all the aforementioned challenges and fabricate highly efficient and stable PSCs. Among the additive engineering strategies, chloride-based additive engineering has become a widely adopted strategy in perovskite film fabrication, with methylammonium chloride (MACl) serving as the most widely employed additive across numerous reports.^36^ MACl facilitates the growth of an initial chloride-rich intermediate phase, resulting in stabilizing the photoactive phase of the perovskite along with significantly increased grain size, enhanced crystallinity, and improved surface coverage, helping to eliminate pinholes.^37,38^ In mixed-halide systems, MACl addition has been shown to induce more effective halide homogenization, which suppresses light-induced halide phase segregation (a major instability factor).^39,40^ Despite its benefits, the usage of MACl is associated with its own critical challenges. The high volatility of MACl introduces multiple detrimental pathways during film formation. Recent studies have shown that MACl does not simply volatilize benignly during annealing; instead, MACl undergoes deprotonation to neutral methylamine (MA^0^) and HCl; the released MA^0^ then can react chemically with formamidinium cations to form *N*-methyl formamidinium iodide (MFAI), predominantly as cis/trans isomers, which can further react with PbI_2_ to yield a yellow 2H-phase *cis*-MFAPbI_3_ perovskite.^41,42^ Such side reactions, combined with the nonsynchronous sublimation of MACl and its reaction products, are expected to create local nonstoichiometry and halogen vacancies, providing trap states that facilitate nonradiative recombination and strongly increase sensitivity to processing conditions.^43,44^ This volatility-driven instability renders the crystallization process highly sensitive to minor environmental fluctuations—including temperature gradients, humidity variations, and substrate thermal mass differences—leading to severe sample-to-sample variability and reproducibility challenges that hinder large-scale fabrication.^45,46^ Alternative chloride sources, such as formamidinium chloride (FACl), propylammonium chloride (PACl),^47^ and chloride additives based on stoichiometric MACl/PbCl_2_ precursors (often described as MAPbCl_3_-type routes),^48^ have been explored. Despite these developments, a systematic head-to-head comparison of MACl and MACl/PbCl_2_-derived chloride additives (MCP) in identical MCMH perovskites is lacking, particularly mechanistic investigations into how the chloride delivery pathway—rather than merely the presence of chloride—affects crystallization dynamics, reproducibility, and phase homogeneity. This knowledge gap hinders the rational selection of additives for compositionally complex perovskite systems.

This study presents a systematic investigation of the effects of chloride additives (MACl and MCP) on MCMH PSCs, with copper thiocyanate (CuSCN) as the inorganic hole-transport layer (HTL). We conducted a batch-level statistical analysis of 25 films for each condition (control, MACl-20, and MCP-5), fabricated across several independent batches on different days from freshly prepared precursor solutions, revealing that the addition of MACl—contrary to expectations in the existing literature—induces severe photoluminescence (PL) quenching in the majority of films. This quenching indicates the formation of defect-induced nonradiative recombination centers and phase inhomogeneity. These effects manifest as X-ray diffraction (XRD) (200) peak splitting and a secondary PL emission peak near 815 nm, observed immediately after fabrication without light exposure, suggesting a crystallization-origin mechanism distinct from the well-documented light-induced halide phase segregation. We demonstrate that MCP serves as an effective alternative chloride source, successfully eliminating phase inhomogeneity. Films modified with MCP exhibit phase-pure perovskite, an approximately one-order-of-magnitude increase in PL intensity, a ∼7–9-fold extension of carrier lifetime (from ∼180 ns to ∼1.6 μs), and a ∼20% relative improvement in device power conversion efficiency (PCE). Our results establish that the chloride delivery pathway plays a critical role in governing phase homogeneity within mixed-halide systems and offer practical guidelines for additive selection in perovskite fabrication.

## Experimental Methods

### Materials

Lead iodide (PbI_2_), lead bromide (PbBr_2_), and lead chloride (PbCl_2_), along with cesium iodide (CsI), copper thiocyanate (CuSCN), and solvents such as *N*,*N*-dimethylformamide (DMF), dimethyl sulfoxide (DMSO), diethyl ether (DEE), and diethyl sulfide, were obtained from Sigma-Aldrich. Tin­(IV) oxide (SnO_2_), containing 15 wt % in an aqueous colloidal dispersion, was procured from ThermoFisher (Germany). Additionally, formamidinium iodide (FAI) and methylammonium chloride (MACl) were supplied by Greatcell Solar Materials (Australia). Furthermore, indium tin oxide (ITO) glass substrates were obtained from Visiontek Systems Ltd. (United Kingdom).

### Substrate Preparation

ITO glass substrates were chemically etched using zinc powder and diluted hydrochloric acid (HCl). The substrates were ultrasonically cleaned sequentially in a deionized water-acetone mixture (30 min), followed by acetone, ethanol, and isopropanol (15 min each). After drying under nitrogen flow, substrates were treated with UV-ozone for 20 min prior to device fabrication.

### Electron Transport Layer (ETL)

Commercial SnO_2_ colloidal dispersion (15 wt % in H_2_O) was diluted with deionized water (1:3 v/v) and stirred at room temperature for 2 h.

### Perovskite Precursor

The perovskite precursor FA_0.9_Cs_0.1_Pb­(I_0.9_Br_0.1_)_3_ was synthesized through dissolution of formamidinium iodide (FAI, 1.26 M), cesium iodide (CsI, 0.14 M), lead iodide (PbI_2_, 1.19 M), and lead bromide (PbBr_2_, 0.21 M) in a mixed solvent of dimethylformamide (DMF) and dimethyl sulfoxide (DMSO) at a volume ratio of 4:1. Methylammonium chloride (MACl) was incorporated at molar concentrations ranging from 10 to 40 mol % relative to the total Pb^2+^ content. Additionally, a supplementary MCP precursor was prepared by dissolving methylammonium chloride (MACl) and lead chloride (PbCl_2_) at a 1:1 molar ratio in a DMF:DMSO solvent mixture (4:1 volume ratio). All precursor solutions were maintained under continuous stirring at room temperature for a duration of one night. Subsequently, 5 mol % and 10 mol % of the MCP solution were incorporated into the baseline FA_0.9_Cs_0.1_Pb­(I_0.9_Br_0.1_)_3_ precursor (prepared without MACl). The resulting combined precursor system was stirred for an additional 30 min at room temperature and then filtered through a 0.22 μm PTFE membrane filter.

### Passivation and Hole Transport Layer (HTL) Solutions

The octylammonium iodide (OAI) passivation solution was prepared by dissolving OAI (2 mg) in isopropanol (1 mL). The CuSCN HTL was prepared by dissolving CuSCN (30 mg) in diethyl sulfide (1 mL) under stirring at room temperature for 4 h.

### Device Fabrication

The SnO_2_ solution was spin-coated onto the ITO substrate at 3000 rpm for 30 s, followed by annealing at 150 °C for 30 min under controlled humidity (35% RH). The FA_0.9_Cs_0.1_Pb­(I_0.9_Br_0.1_)_3_ perovskite solution was spin-coated onto the ETL at 1000 rpm for 5 s, followed by 4000 rpm for 30 s, and 800 μL of DEE was dispensed as the antisolvent 15 s before spin-coating completion. Both control (additive-free) and MCP-processed samples were annealed at 100 °C for 30 min inside a nitrogen-filled glovebox, while MACl-processed samples were annealed at 150 °C for 10 min under ambient atmosphere (RH ∼ 30%). The OAI passivation layer was deposited by spin-coating at 3000 rpm for 30 s, followed by annealing at 100 °C for 5 min. Subsequently, CuSCN was spin-coated at 2000 rpm for 30s and annealed at 80 °C for 10 min. Finally, gold (Au) electrodes (100 nm) were deposited by thermal evaporation under vacuum at ∼10^–6^ mbar.

### Characterization Methods

#### Structural and Morphological Analysis

X-ray diffraction (XRD) patterns were recorded using a Bruker D8 Advance diffractometer equipped with a Cu Kα radiation source (λ = 0.154 nm), a Kα1 monochromator, and a LynxEye detector (Model A17-B60). Surface topography was analyzed by Atomic Force Microscopy (AFM) using a Bruker IconXR system in noncontact mode; subsequent surface analysis and processing were performed using Gwyddion software.

#### Optical Properties

UV–visible absorbance spectra were acquired using an Agilent Technologies Cary 300 spectrometer. Steady-state and time-resolved photoluminescence (PL) measurements were conducted using an Edinburgh Instruments FLS980 spectrometer. The PL decay kinetics were analyzed using the built-in Fluorescence Analysis Software Technology (FAST) package. The photoemission yield spectroscopy in air (PYSA) was conducted using the AC-2S Photoemission Yield Spectroscopy in Air.

#### Electrical and Device Characterization

Current density–voltage (*J*–*V*) characteristics were measured using a Keithley 2400 source meter in a two-probe configuration. The devices were mounted on a Linkam HFS600E stage and measured at room temperature in ambient air. The solar simulator was a NEWPORT 94082A model based on a 1600 W xenon lamp and equipped with an AM1.5G filter. Conventional spectral mismatch correction was carried out using a certified reference silicon photodetector, with the spectral response of the test cells extracted from external efficiency measurements made with ENLITECH QE-T equipment. Statistical analysis of device performance was conducted across several independent batches of 9 devices processed under similar conditions.

## Results and Discussion

### Microstructural and Crystallographic Inhomogeneity

Methylammonium chloride (MACl) is widely used as an additive in perovskite solar cells to promote grain growth and improve efficiency. However, concerns persist regarding compositional heterogeneity and the reproducibility of performance in films processed with MACl. To address these issues and evaluate alternatives, we systematically compared the MACl additive with MCP chloride additive, obtained from a stoichiometric MACl/PbCl_2_ precursor, which acts as a nonvolatile chloride source that decomposes at specified temperatures instead of volatilizing randomly. We prepared MCMH perovskite films with the nominal composition FA_0.9_Cs_0.1_Pb­(I_0.9_Br_0.1_)_3_ on glass substrates. We incorporated MACl at 10, 20, 30, 35, and 40 mol % relative to Pb^2+^ (designated as MACl-10 through MACl-40), and added MCP at 5 and 10 mol % (MCP-5 and MCP-10). Control films without any additives and MCP-processed films underwent annealing at 100 °C for 30 min, whereas MACl-processed films underwent annealing at 150 °C for 10 min (see Experimental Methods for details). For MACl-processed films, we adopted the annealing protocol reported in the literature (150 °C for 10 min in ambient atmosphere with ∼30% RH). Shen et al.^39^ showed that MACl-processed wide-bandgap perovskite films require higher annealing temperatures and benefit from annealing in humid air to drive off excess MACl/MA and maximize PL and device performance, and our protocols were therefore chosen as individually optimized processing routes for MACl-processed and control/MCP-processed films. We characterized the films using photoluminescence (PL) spectroscopy, X-ray diffraction (XRD), Williamson–Hall (W–H) and Halder–Wagner (H–W) line-profile analysis, atomic force microscopy (AFM), power spectral density (PSD) analysis, and photoemission yield spectroscopy in air (PYSA) to assess crystallinity, microstructure, morphology, and ionization potential.

### Spectroscopic Evidence for Compositional Heterogeneity

To investigate how MACl and MCP additive concentrations influence defect passivation, suppression of nonradiative recombination, and the emergence of phase inhomogeneity across the explored composition range, we performed steady-state photoluminescence (PL) spectroscopy on the corresponding perovskite thin films. Figure 1a shows representative PL spectra for the control, MACl-10, MACl-20, MACl-30, MACl-35, MACl-40, and MCP-5 films, all measured under identical excitation conditions and normalized by the corresponding film absorbance at excitation wavelength to enable a shape-based comparison of the emission spectra. The control sample exhibits a sharp emission peak centered at approximately 780 nm (1.59 eV), consistent with the expected bandgap of FA_0.9_Cs_0.1_Pb­(I_0.9_Br_0.1_)_3_. In contrast, the MACl-processed films show highly variable PL responses relative to the control, with some compositions showing pronounced PL quenching and others exhibiting PL enhancement, indicating poorly controlled or irreproducible film formation. This behavior differs from previous reports, which generally show 2–5-fold PL enhancement and increased PL quantum yield upon MACl addition, attributed to grain-boundary passivation and defect density.^39,49^

**Figure 1:**
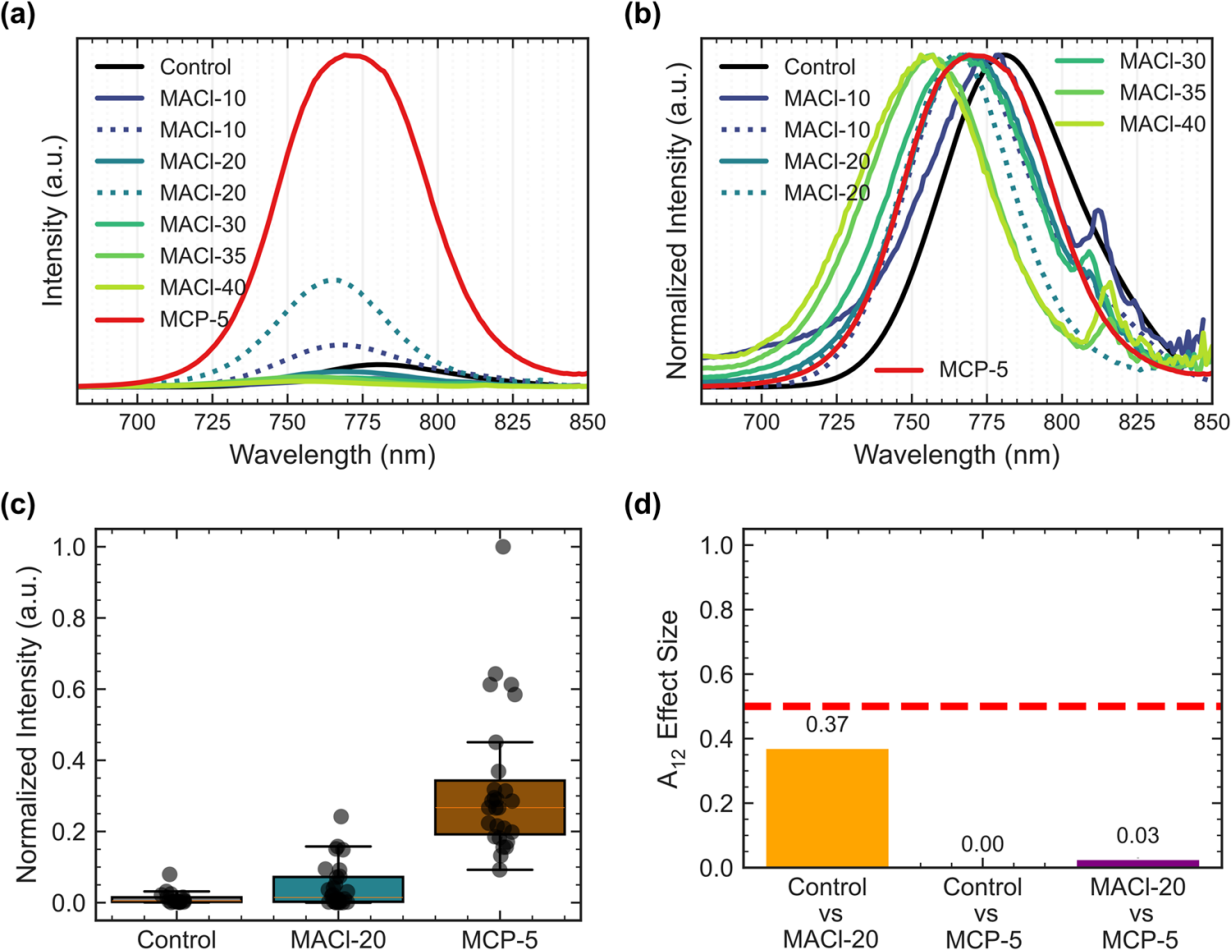
PL characterization of control, MACl-20, and MCP-5 perovskite films. (a) Representative PL spectra normalized with respect to the absorption at the excitation wavelength, enabling direct comparison of emission yields across samples. (b) The same absorption-corrected PL spectra normalized to their respective maximum peak intensities, highlighting spectral shape and peak position differences. (c) Statistical boxplot of the raw PL peak intensities for control, MACl-20, and MCP-5 films (*n* = 25 samples per category), illustrating the statistical distribution and reproducibility of emission across independently fabricated samples. (d) Varga–Delaney *A*_12_ effect sizes quantifying the stochastic superiority of pairwise sample comparisons based on the raw PL peak intensities, where *A*_12_ > 0.5 indicates a higher probability of the first group exhibiting greater raw PL peak intensity than the second.

Figure 1b shows that, in addition to the primary emission peak, all MACl-processed films exhibit a secondary emission feature at around 810–815 nm. Such secondary PL emission peaks are commonly associated with the formation of iodide-rich domains in mixed-halide perovskites and are generally interpreted as evidence of halide phase segregation within the film.^50^ In contrast, the MACl-processed films whose PL intensity exceeds that of the control do not display this secondary emission feature.

In sharp contrast, the MCP-5-processed film exhibits a single, narrow PL peak with an intensity approximately 1 order of magnitude higher than that of the control and shows no detectable secondary emission. These divergent behaviors suggest that MACl incorporation tends to induce compositional heterogeneity during crystallization, whereas MCP incorporation promotes the formation of a more compositionally uniform, highly emissive perovskite phase. The observed spectroscopic signatures are consistent with the coexistence of compositionally distinct domains—likely iodide-rich regions and chloride/bromide-enriched regions—arising from nonequilibrium crystallization processes associated with MACl volatilization. Because the MACl-20 and MCP-5 films were fabricated using distinct annealing protocols, their comparison is more appropriately interpreted as a comparison between two additive-specific processing routes, rather than as a fully isolated assessment of additive chemical identity. To probe the influence of annealing atmosphere within the MACl-assisted route, we prepared additional MACl-20 films annealed at 150 °C for 10 min in a N_2_ glovebox and recorded their steady-state photoluminescence (PL) spectra (Figure S1), normalized by the corresponding film absorbance at the excitation wavelength. These samples exhibit PL characteristics analogous to those of MACl-20 films annealed in air, including substantial sample-to-sample variability in PL intensity and spectral line shape. This observation indicates that the heterogeneous PL response of MACl-20 films is not eliminated by simply substituting an N_2_ atmosphere for air during annealing. However, because we did not implement a full factorial processing design in which each composition is systematically examined across multiple annealing temperatures, times, and atmospheres, the observed differences cannot be unambiguously ascribed to the intrinsic chemical identity of MACl alone. Consequently, we avoid attributing all observed heterogeneity solely to MACl and instead frame our discussion in terms of MACl-20- and MCP additive-processing routes, evaluated under the processing conditions that are commonly employed and experimentally optimized for each additive in this work.

To further clarify the chemical role of the MCP additive, we prepared additional films in which MACl and PbCl_2_ powders (1:1 molar ratio) were added directly to the perovskite precursor without precomplexation (denoted MACl + PbCl_2_). These films were processed under the same conditions as the MCP-5 samples, and their steady-state PL spectra were recorded (Figure S2) and normalized by the corresponding film absorbance at the excitation wavelength. Within experimental uncertainty, the PL spectra and intensities of MACl + PbCl_2_-processed films are very similar to those of MCP-5 films, indicating that the key optoelectronic behavior is governed by the presence of a nonvolatile MA–Pb–Cl chloride source, rather than by the formation of a distinct crystalline MCP phase prior to or during film deposition.

### Intensity Analysis and Statistical Implications

The compositional heterogeneity observed in Figure 1a,1b was found to be independent of MACl concentration, suggesting that this instability cannot be mitigated through concentration optimization and may be an intrinsic characteristic of the MACl crystallization pathway. In addition, small sample sizes (*n* = 3–5 per concentration) may obscure subtle trends or miss a narrow optimal window. To rigorously assess reproducibility, we conducted a large-sample statistical investigation comprising *n* = 25 films each for the control, MACl-20, and MCP-5 conditions, with PL characterization performed on films fabricated in several independent batches (typically 5–6 films per batch on different days from freshly prepared precursor solutions) (see Figure S3). This design allows a direct comparison of sample-to-sample and batch-to-batch variability between MACl-20 and MCP-5 processing. The resulting distributions of raw PL peak intensities were analyzed using nonparametric statistics, bootstrap resampling, and a quantile-based method^51,52^ to quantify the impact of chloride-based additives on compositional control and optical quality (Figure S4).

Figure 1c shows comparative box plots of the raw PL peak-intensity distributions. The control films exhibit the lowest PL emission, reflecting substantial nonradiative recombination losses. In contrast, incorporation of chloride-based additives yields a marked enhancement in radiative yield. To statistically validate these observations without assuming a Gaussian distribution, we applied Mann–Whitney U tests (see Table S1). The MCP-5-processed films demonstrate complete stochastic dominance over the control in this sample set (*U* = 0.0, *p* = 1.92 × 10^–8^ and *r* = 0.839), with a maximal Vargha–Delaney effect size of *A*_12_ (Control, MCP-5) ≈ 0.0, indicating that the probability that a randomly selected control sample exhibits higher raw PL intensity than a randomly selected MCP-5 sample is effectively zero (Figure 1d). This result (*p* < 0.001 and *A*_12_ (Control, MCP-5) ≈ 0) supports the conclusion that the optoelectronic enhancement with MCP-5 is systematic rather than attributable to isolated high-performing outliers.

The statistical analysis of MACl-20 films reveals a more complex passivation behavior. Standard rank-based testing shows substantial overlap with the control distribution (*U* = 187, *p* = 0.147, and *r* = 0.216), with a moderate Vargha–Delaney effect size of A_12_ (Control, MACl-20) ≈ 0.371. However, bootstrap resampling (see Figure S4a and Tables S2 and S3) reveals a statistically significant increase in mean raw PL intensity for MACl-20 relative to the control (*p* = 0.026), driven by a subset of high-emission films. This discrepancy between median-based and mean-based analyses indicates that MACl-20 is heterogeneous: the additive drives substantial improvements in a subset of films while leaving others comparable to or below the control baseline. For completeness, we also compared MACl-20 and MCP-5 directly. The Mann–Whitney test yields *U* = 20.0, *p* = 1.75 × 10^–9^, *r* = 0.813, with a Vargha–Delaney effect size *A*_12_ (MACl-20, MCP-5) ≈ 0.03, indicating that the probability that a randomly selected MACl-20 film exhibits higher raw PL intensity than a randomly selected MCP-5 film is only ∼3%. This confirms that, within our sample set, MCP-5 not only outperforms the control but also systematically outperforms MACl-20. Hence, MCP-5 functions as a more uniform passivator in our experiments, suppressing nonradiative recombination pathways uniformly across all tested films.

To further elucidate how these additives influence fabrication yield, we analyzed the raw PL peak intensity at discrete quantiles (10th to 90th percentiles), as shown in Figure S4b. In this framework, lower quantiles correspond to samples with lower PL intensity, indicative of higher nonradiative recombination losses within each condition, whereas higher quantiles correspond to samples with superior optoelectronic quality. Negligible emission in the lower quantiles indicates that a substantial fraction of samples fails to achieve adequate radiative recombination, reflecting poor process reproducibility, whereas strong emission confined to the higher quantiles suggests that only a small subset of samples attains functional optoelectronic quality. The control group exhibits negligible PL emission across most of the distribution (10th–75th percentiles), with only the 90th percentile showing a measurable signal, indicating that the baseline crystallization window is narrow and highly susceptible to processing variations. While MACl-20 shows virtually no improvement at the lower quantiles (10th–50th percentiles), meaningful improvement is observed only at the 75th and 90th percentiles, indicating that this additive amplifies quality differences across the distribution rather than uniformly elevating performance. In contrast, MCP-5 functions as a robust process stabilizer, inducing substantial radiative emission across all quantiles—including the 10th and 25th percentiles, where control and MACl-20 films exhibit minimal emission. This behavior indicates that MCP-5 reduces the impact of stochastic processing variations, ensuring that even samples at the lower end of the performance distribution achieve functional optoelectronic quality and thereby significantly enhancing overall fabrication yield and process reproducibility. The quantile analysis also provides critical insight into the variance structure of these distributions. Although the MCP-5-processed films exhibit the highest statistical variance, as evident from the box plot, the quantile data show that this variance is not associated with poor reproducibility. Notably, the 10th percentile raw PL intensity of the MCP-5 films exceeds the 90th percentile of the control films by a factor of approximately six, meaning that even the worst-performing MCP-5 samples substantially outperform the best-performing control samples.

### Structural Origins of Compositional Heterogeneity

Motivated by the PL evidence for MACl-induced heterogeneity, we performed XRD measurements on perovskite films prepared with varying MACl (0–40 mol %) and MCP (5 and 10 mol %) additive concentrations to verify the perovskite phase and assess structural inhomogeneity. Figures 2a and S5 show the corresponding XRD patterns. All films exhibit the characteristic diffraction peaks of the cubic phase of the perovskite FA_0.9_Cs_0.1_Pb­(I_0.9_Br_0.1_)_3_ at 2θ ≈ 14.1°, 28.4°, and 43.1°, indexed to the (100), (200), and (300) planes. The MACl-processed films displayed progressively enhanced (100) and (200) peak intensities accompanied by a systematic reduction in full width at half-maximum (FWHM; Figure 3a), from 0.139° (control) to 0.080° (MACl-10) to 0.061° (MACl-40). Consistent with the Scherrer relationship,^53^ this peak narrowing reflects an increase in crystallite size.

**Figure 2:**
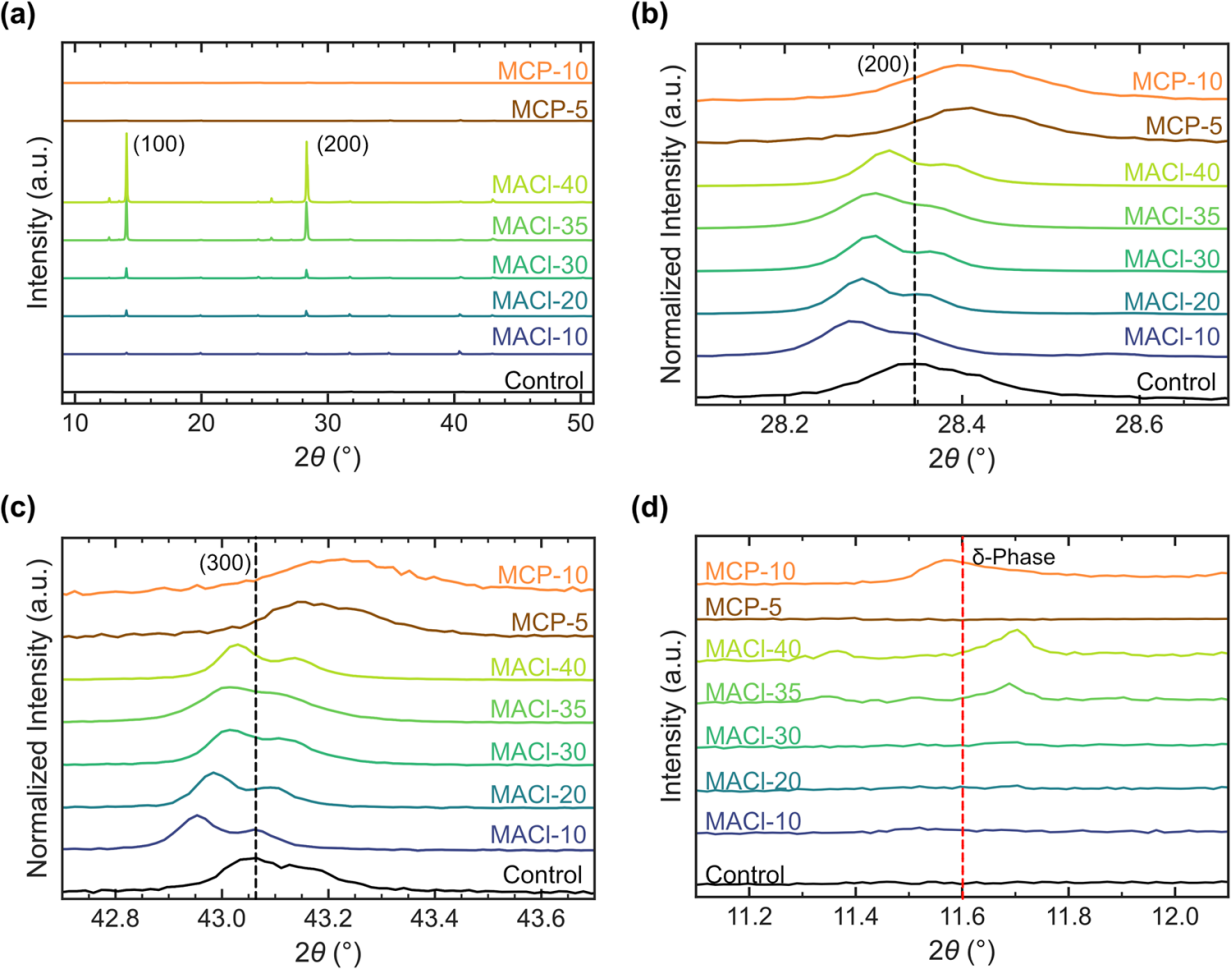
Structural evolution and crystallographic analysis of perovskite films as a function of additive concentration: (a) XRD patterns, (b) XRD patterns tracking the evolution of (200) peak, (c) XRD patterns tracking the evolution of (300) peak, and (d) evolution of δ-phase.

**Figure 3:**
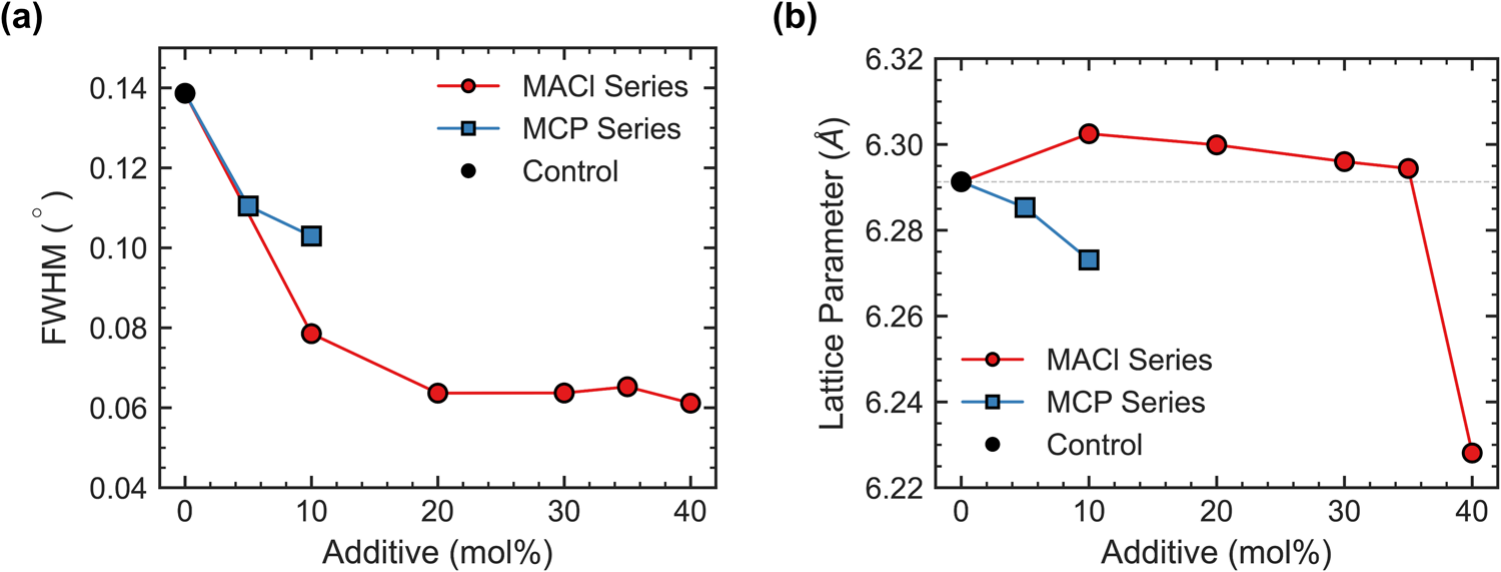
Quantitative crystallographic parameters extracted from XRD analysis of FA_0.9_Cs_0.1_Pb­(I_0.9_Br_0.1_)_3_ perovskite films as a function of additive concentration. (a) Evolution of FWHM of the primary perovskite reflection. The decrease in FWHM with increasing MACl concentration indicates peak narrowing, consistent with increased crystallite size. (b) Evolution of the average lattice parameter calculated from the main perovskite reflections. The dashed horizontal line indicates the lattice parameter of the control film.

However, closer zoomed inspection reveals critical structural heterogeneity. While control and MCP-processed films display a single, symmetric (200) peak at 2θ ≈ 28.4°, all MACl-processed films exhibit a characteristic doublet (Figure 2b): primary peak at ≈28.4° and secondary component at ≈28.3°. A similar doublet is also present at the (300) peak. This peak splitting is widely recognized as a signature of compositional phase segregation into iodide-rich and bromide-/chloride-enriched domains in mixed-halide perovskites.^54–56^ In contrast to previously reported photoinduced segregation,^54–56^ the doublet in our MACl-processed films is already present in the as-prepared state and thus reflects MACl-driven nonequilibrium crystallization rather than light-induced effects. The lower-angle component corresponds to an iodide-rich phase with an expanded lattice parameter (*a* ≈ 6.31 Å), consistent with previous reports that associate iodide-enriched domains with larger lattice constants and red-shifted PL emission in mixed-halide perovskites.^50,54,57^ We therefore assign this phase as the structural origin of the secondary PL peak at ∼810–815 nm (Figure 1b).

Analysis of the (111) reflection (2θ ≈ 24.5°) provides complementary evidence (Figure S6d). The control film shows a normal, symmetric (111) peak, whereas at intermediate MACl concentrations (MACl-10 to MACl-30) asymmetric broadening with a lower-angle shoulder emerges, confirming the presence of two coexisting phases with disparate lattice parameters. At higher MACl concentrations (MACl-35 and MACl-40), the (111) intensity is dramatically suppressed due to strong (100)/(200) preferential orientation,^39,45^ such that the (111) planes become perpendicular to the substrate and therefore contribute only weakly in the used θ–2θ geometry.^58^ In contrast, MCP-5 and MCP-10 films exhibit a single, well-defined (100), (111), (200), and (300) peaks that are coherently shifted to higher angles relative to the control, consistent with a small lattice contraction expected from partial chloride incorporation into the mixed-halide perovskite lattice.

Average cubic lattice parameters (denoted *a*) were calculated from the (100), (200), and (300) peaks using Bragg’s law (Figure 3b). The absolute changes are small, but within the MACl-processed series, the lattice parameter lies slightly above the control (*a* ≈ 6.30–6.32 Å for MACl-10 to MACl-35 vs ≈6.29 Å for the control; Figure 3b). Together with the resolved doublets at the (111), (200), and (300) peaks (Figures 2b,c and S6d), this modest expansion is most naturally

explained as the weighted average of coexisting bromide/chloride-enriched and iodide-enriched domains with distinct lattice constants, in agreement with the bimodal PL response. At MACl-40, an unusual nonlinear behavior emerges: the lattice parameter abruptly contracts to 6.23 Å, consistent with chloride incorporation (Cl^–^ = 181 pm).^59^ Despite this contraction, peak splitting persists, indicating a complex phase-separated state in which chloride-rich/bromide-rich and iodide-rich domains coexist, thereby demonstrating the unpredictable nature of high-concentration MACl processing.

In stark contrast, MCP-processed films exhibit controlled structural evolution. The XRD (100) peak FWHM decreases moderately (0.139° to 0.110° for MCP-5 and 0.101° for MCP-10), and the cubic lattice parameter contracts smoothly from 6.29 Å (control) to 6.28 Å (MCP-5) and 6.27 Å (MCP-10), consistent with systematic chloride incorporation. Critically, no peak splitting is observed in the (111), (200), or (300) reflections of MCP-processed films (Figures 2b,2c and S6d), confirming the absence of compositional segregation. Low-angle XRD analysis (12°–13°) reveals PbI_2_ at 2θ ≈ 12.7° in all films (Figure S6b); the integrated intensity increased with MACl concentration, suggesting residual PbI_2_ accumulation at grain boundaries following MACl volatilization during annealing.^43,60^ MCP-5 showed minimal PbI_2_, indicating compositional purity, whereas MCP-10, MACl-35, and MACl-40 exhibit additional secondary phases (δ-phase at 11.6°, and an unidentified phase at 12.3°), indicating lattice saturation and segregation into nonperovskite polymorphs (Figure 2d). This behavior establishes the solubility limit for MCP in the FA/Cs-based system and rationalizes the selection of MCP-5 as the optimal.

### Williamson–Hall Analysis: Decoupling Size and Strain

To decouple crystallite size and lattice strain contributions to XRD peak broadening, Williamson–Hall (W–H) and Halder–Wagner (H–W) line-profile analyses were performed on control, MCP-processed, and MACl-processed films. The resulting W–H plots (β cos θ vs 4 sin θ) and H–W plots ((β*/ d*)^2^ vs β*/(d*)^2^) are shown in Figures S7–S10. However, instrumental broadening analysis reveals fundamental limitations in applying these methods to MACl-processed samples because extreme grain coarsening pushes the peak widths toward the resolution limit of the laboratory diffractometer.^61^ FWHM values (β) were extracted from (100), (200), and (300) peaks and corrected for instrumental broadening (β_inst_) determined from a Si NIST standard, yielding sample-specific broadening (β_sample_) via Gaussian deconvolution: β_sample_^2^ = β_obs_^2^ – β_inst_^2^. The reliability of line-profile analysis critically depends on the ratio β_sample_/β_inst_, with values less than 1 indicating that instrumental effects dominate sample contributions, leading to unacceptably high relative errors in extracted parameters.^61–63^ Table S4 summarizes instrumental broadening analysis for representative samples. Control and MCP-processed films exhibit adequate signal-to-noise ratios and β_sample_/β_inst_ > 1 for multiple reflections, enabling reliable W–H/H–W fits. In stark contrast, all MACl-processed samples show β_sample_/β_inst_ < 1 at the (100) reflection. The extremely narrow XRD peaks in MACl samples—particularly at (100) where β_sample_ = 0.032° – 0.035°—correspond to apparent grain sizes exceeding 250–300 nm, i.e., approaching or surpassing the XRD coherence length where laboratory diffractometers can no longer reliably extract crystallite size and microstrain from peak broadening.^64^ This instrumental resolution limit is a direct consequence of successful MACl-mediated Ostwald ripening: MACl clearly drives substantial grain coarsening, but at the same time it precludes conventional W–H/H–W analysis of size-strain separation in these samples.

Control and MCP-5 films exhibit well-behaved linear responses with high fit quality (*R* ^2^ > 0.92 for W–H; *R* ^2^ > 0.98 for H–W). The control film exhibits microstrain ε = 0.115% (W–H) and 0.397% (H–W), while MCP-5 shows comparable values (ε = 0.128% W–H, 0.390% H–W) with *R* ^2^ = 0.932 and 0.990, respectively. Positive W–H *y*-intercepts and positive H–W slopes indicate that uniform crystallite size distribution and homogeneous strain assumptions are satisfied for these samples.^65,66^ In contrast, MACl-processed films exhibit systematic deviations from ideal behavior. W–H analysis yields high-quality linear fits (*R* ^2^ = 0.930–0.999) across all MACl concentrations; however, *y*-intercepts are negative, indicating that peak broadening is dominated by microstrain rather than finite crystallite size. This conclusion is, however, partly artifactual: when sample broadening at low-angle reflections is dominated by instrumental contribution (β_sample_/β_inst_ < 1), small uncertainties in instrumental correction (β_inst_correction_) propagate into large relative errors in β_sample_ and thus in the extracted size-strain parameters. Even with this caveat, the apparent microstrain from W–H fits increases monotonically from ε = 0.132% (MACl-10) to 0.222% (MACl-35), indicating progressive lattice distortion despite grain coarsening, while at MACl-40, microstrain decreases abruptly to ε = 0.191%—a nonmonotonic evolution mirroring the lattice parameter contraction observed in Figure 4a.

**Figure 4:**
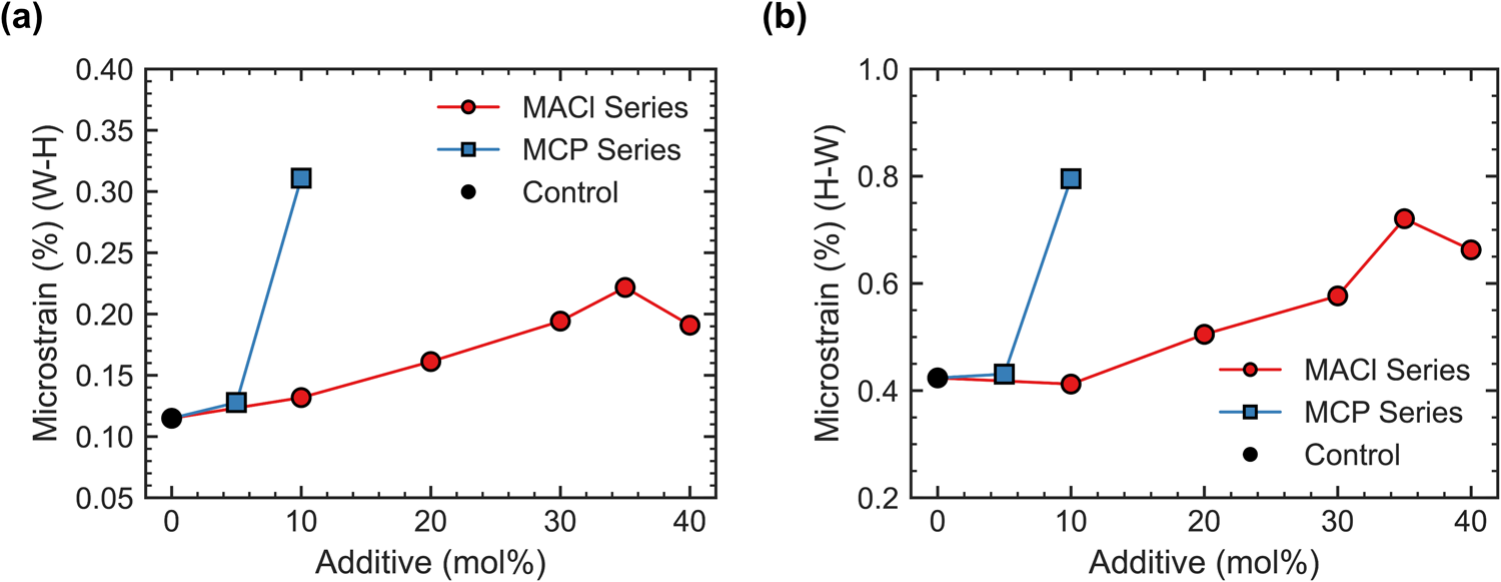
Microstrain analysis of FA_0.9_Cs_0.1_Pb­(I_0.9_Br_0.1_)_3_ perovskite films as a function of additive concentration. Microstrain values were estimated from XRD peak broadening using the (a) W–H method and (b) H–W method. Both analyses indicate an increase in microstrain with increasing additive concentration, with a more pronounced rise observed for the MCP-10 film.

H–W analysis reveals a more severe model breakdown. While control and MCP-5 exhibit normal H–W behavior (positive slopes, *R* ^2^ > 0.98), MACl-20 through MACl-40 films display negative slopes with severely degraded fit quality (*R* ^2^ = 0.041–0.312). Critically, negative H–W slopes can arise from multiple origins: (1) strong crystallographic texture (preferred orientation) creating anisotropic microstrain incompatible with isotropic strain models, or (2) instrumental broadening dominance rendering regression statistically invalid. In MACl-processed films, both factors likely contribute: the strong (100) preferred orientation violates isotropic assumptions, and the extreme grain growth produces instrumental limitations. Apparent H–W microstrain values increase from 0.366% (MACl-10) to 0.636% (MACl-35) before decreasing to 0.300% at MACl-40, a 53% reduction coinciding with the lattice contraction (Figure 4b). This nonmonotonic behavior contrasts with the smooth, monotonic MCP progression (ε = 0.390% to 0.878% for MCP-5 to MCP-10), demonstrating that MACl crystallization exhibits unpredictable concentration-dependent complexity. The agreement of the MCP films with the W–H and H–W models confirms the reliability of the analytical approach and indicates that stoichiometric chloride addition leads to highly uniform microstructures. The evidence for MACl-induced phase segregation is independent of W–H/H–W analysis: (200) peak splitting (Figure 2b) clearly demonstrates coexistence of phases with distinct lattice parameters, while dual PL emission (Figure 1b) directly reveals compositional domains with different bandgaps. The W–H/H–W violations, therefore, reflect analytical limitations when characterizing large-grained, strongly textured films with laboratory XRD, rather than undermining the segregation picture itself.

### Surface Morphology Analysis

Contact-mode atomic force microscopy (AFM) and power spectral density (PSD) analysis were performed to characterize how MACl and MCP additives influence crystallization morphology. PSD analysis separates grain-scale features (low spatial frequencies) from intragrain texture (high frequencies),^67^ enabling identification of phase inhomogeneity and compositional heterogeneity that are not resolvable by XRD, and directly linking surface topology to charge-extraction losses in devices.

Figure S11 shows the 5 × 5 μm^2^ topographic evolution of films with increasing concentrations of MACl and MCP additives. The root-mean-square (RMS) roughness of the control film (no additives) is 19.6 nm. For MACl-processed films, RMS roughness increases monotonically from 20.4 nm (MACl-10) to 25.7 nm (MACl-40), while the MCP-5 film exhibits a reduction to 18.4 nm. In contrast, the MCP-10 film displays a pronounced increase in RMS roughness to 26.9 nm. Among all compositions studied, MCP-5 presents the lowest surface roughness. Although surface smoothness alone does not fully determine photovoltaic performance, it is a crucial geometric requirement for achieving a high fill factor. Reduced RMS roughness enables charge transport layers to form continuous, pinhole-free barriers, thereby maximizing shunt resistance and facilitating efficient interfacial charge extraction.^68,69^

Figures 5d, S12, and S13 show the corresponding grain size distributions as histograms extracted from AFM images, and Table S5 summarizes the grain sizes. The control film exhibits an average grain size of 142 nm with a standard deviation of 86 nm. With MACl incorporation, average grain size increases from 142 nm (control) to 606 nm (MACl-40), while standard deviation expands to 191 nm, indicating broadening of the grain size distribution and sequential nucleation characteristic of heterogeneous crystallization. In contrast, incorporation of MCP does not produce significant changes in grain size relative to the control: the average grain size for MCP-5 films increases slightly to 165 nm, whereas for MCP-10 it remains approximately 143 nm.

**Figure 5:**
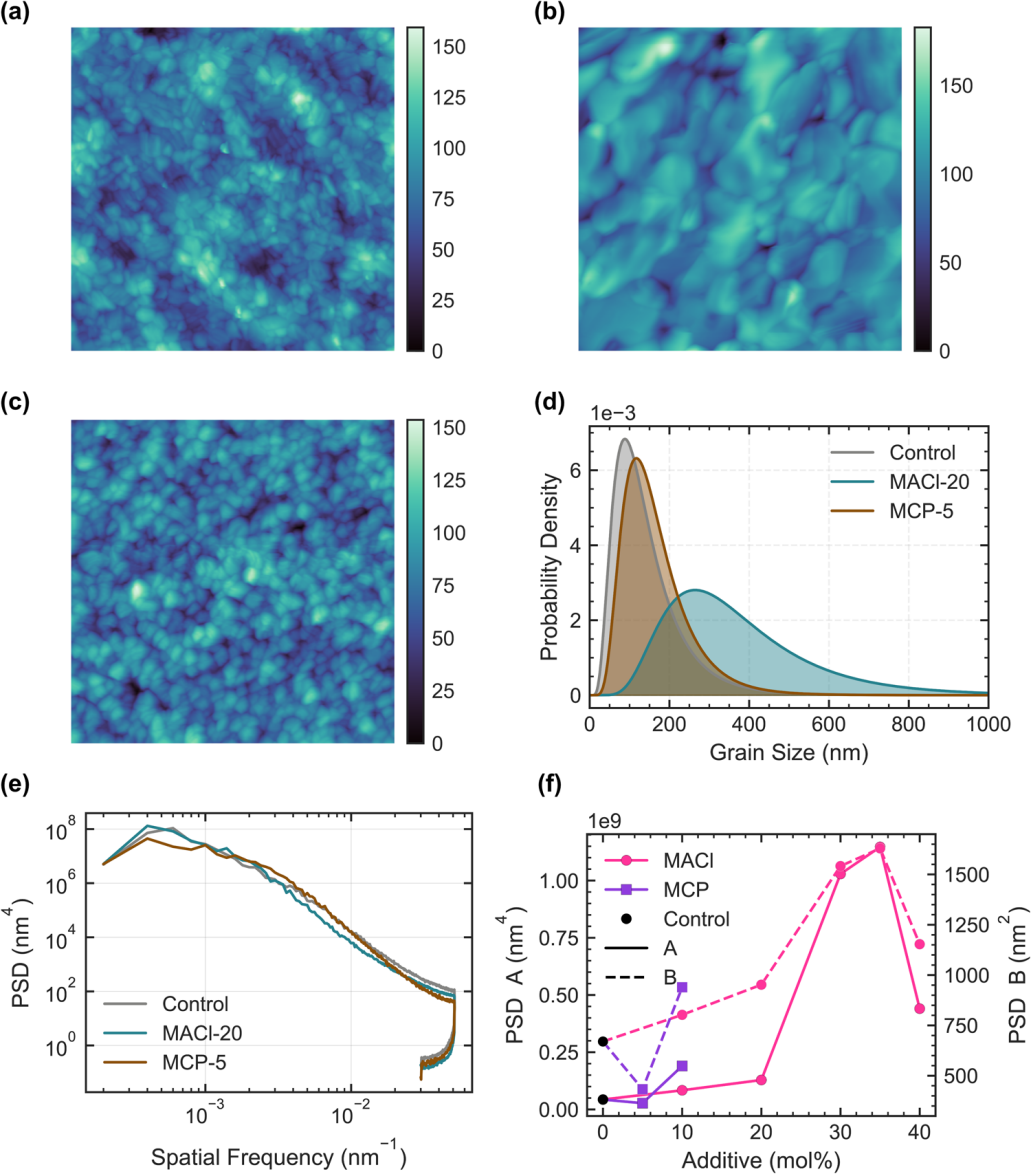
AFM images of perovskite films based on the two chloride additives: (a) Control, (b) MACl-20, (c) MCP-5; (d) Grain size distributions extracted from the AFM images for control, MACl-20, and MCP-5; (e) PSD spectra of the AFM height profiles as a function of spatial frequency and (f) PSD amplitudes A and B as a function of chloride-based additive content.

To quantitatively characterize surface morphology evolution beyond conventional RMS roughness, PSD analysis was performed using the *k*-correlation (ABC) model^67^

$$\mathrm{PS}D_{\mathrm{ABC}}\left(f\right)=\frac{A}{{\left(1+Bf^{2}\right)}^{\left(C+1\right)/2}}$$

where parameter *A* quantifies spectral strength (roughness amplitude), *B* represents the correlation length (characteristic lateral feature size), and *C* describes power-law scaling behavior. This methodology offers a comprehensive analysis of spatial frequency-dependent surface characteristics, facilitating differentiation between beneficial grain coarsening and detrimental heterogeneous crystallization.^67,70^ The full set of PSD curves, fitted ABC parameters, and equivalent RMS values extracted from PSD analysis of 5 × 5 μm^2^ AFM images for all compositions are compiled in Table S6 and Figures 5d, S14, and S15 in the Supporting Information. The control film exhibits parameters *A* = 4.33 × 10^7^ nm, *B* = 669.9 nm, and *C* = 3.1. Addition of 10 mol % MACl increased these parameters to *A* = 8.37 × 10^7^ nm, *B* = 802.5 nm, and *C* = 2.83. This trend is strongly amplified at higher concentrations: for MACl-35, *A* reaches 1.15 × 10^9^ nm (a 24-fold increase), with B extending to 1629.4 nm and *C* = 3.29. The MACl-40 film shows a reduction in *A* to 4.40 × 10^8^ nm, while *B* remains high at 1153.5 nm (*C* = 3.31), suggesting a transition to a distinct morphological regime. In contrast, MCP-processed films maintain substantially lower PSD parameters: MCP-5 yields *A* = 2.74 × 10^7^ nm, *B* = 432.8 nm, and *C* = 3.98—values comparable to or below those of the pristine control—whereas MCP-10 exhibits an increase to *A* = 1.89 × 10^8^ nm, *B* = 939.47 nm, and *C* = 2.99.

The simultaneous increase of spectral strength *A*, correlation length *B*, and RMS roughness with increasing MACl concentration indicates heterogeneous domain formation rather than uniform grain growth. In homogeneous polycrystalline films undergoing uniform grain coarsening, correlation length *B* increases proportionally to grain size while spectral strength *A* remains approximately constant or increases sublinearly, as larger grains redistribute laterally with grain boundary grooves maintaining similar depths.^70,71^ However, MACl-processed samples exhibit superlinear scaling of spectral strength *A* relative to correlation length *B* with MACl concentration, indicating concurrent growth of lateral feature size and amplification of surface-height variance (see Table S6). The correlation length *B* increases from 670 nm (control) to 1629 nm (MACl-35), indicating the lateral domains grown via MACl-mediated Ostwald ripening with a characteristic length of ∼1.6 μm, while the increase in spectral strength A reflects the surface-height contrast between structurally and compositionally distinct regions. Importantly, PSD-derived correlation lengths provide independent validation of grain sizes where XRD line-broadening analysis reaches instrumental limits. For MACl-processed samples, the increase in *B* is consistent with XRD peaks approaching the instrumental resolution limit, confirming crystallite sizes approaching or exceeding the micrometer scale. This cross-technique validation demonstrates that MACl-mediated Ostwald ripening successfully produces large-grain-sized films while simultaneously generating compositional heterogeneity that manifests as elevated surface roughness and broad, superlinearly scaled PSD parameters. Conversely, MCP-5 maintains low PSD parameters comparable to control films, indicating homogeneous integration without compositional inhomogeneity and reflecting direct A-site/halide incorporation rather than MACl’s volatile behavior, which can create heterogeneity.

The evolution of the PSD parameters *A* and *B* as a function of additive concentration is summarized in Figure 5f, with the corresponding fitted values listed in Table S6. Together, these data show that MACl-assisted processing leads to strong, concurrent increases in spectral strength *A* and correlation length *B* with increasing MACl content, whereas MCP-5 retains values comparable to or smaller than the control, and MCP-10 shows only a moderate increase. We note that these trends are derived from PSD analysis of 5 × 5 μm^2^ AFM images and thus reflect morphology over the lateral length scales probed in this study.

### Optoelectronic Properties

To elucidate charge carrier recombination pathways and quantify the effectiveness of chloride-based additives in defect passivation, time-resolved PL (TRPL) spectroscopy was performed on control, MACl-20, and MCP-5-processed films (Figure 6a,b). The TRPL curves were fitted using the biexponential decay equation *y*(*t*) = *y*_0_ + *A*_1_ exp­(−*t*/τ_1_) + *A*_2_ exp­(−*t*/τ_2_), where τ_1_ and τ_2_ represent the fast and slow decay process time constants, and *A*_1_ and *A*_2_ represent corresponding amplitudes for each component. The average carrier lifetime was calculated by using the equation τ_ave_ = (*A*_1_τ_1_^2^ + *A*_2_τ_2_^2^)/(*A*_1_τ_1_ + *A*_2_τ_2_). The fitting parameters and calculated carrier lifetime values are summarized in Table S7. The control film exhibits τ_1_ = 7.1 ns and τ_2_ = 210.5 ns, giving an average lifetime of τ_avg_ ≈ 182 ns, while MACl-20 already increases these components to τ_1_ = 113.5 ns and τ_2_ = 877.1 ns (τ_avg_ ≈ 820 ns). For MCP-5, both τ_1_ and τ_2_ are further extended to 442.5 and 1487.3 ns, respectively, resulting in τ_avg_ ≈ 1335 ns. The higher decay components indicate that deposited perovskite films retain a larger carrier population at long delay times, attributed to strong suppression of trap-assisted nonradiative recombination and improved bulk ordering.^49^ Taken together, the extended decay components and markedly enhanced steady-state PL intensity of MCP-5 films are therefore consistent with strongly suppressed nonradiative recombination.

**Figure 6:**
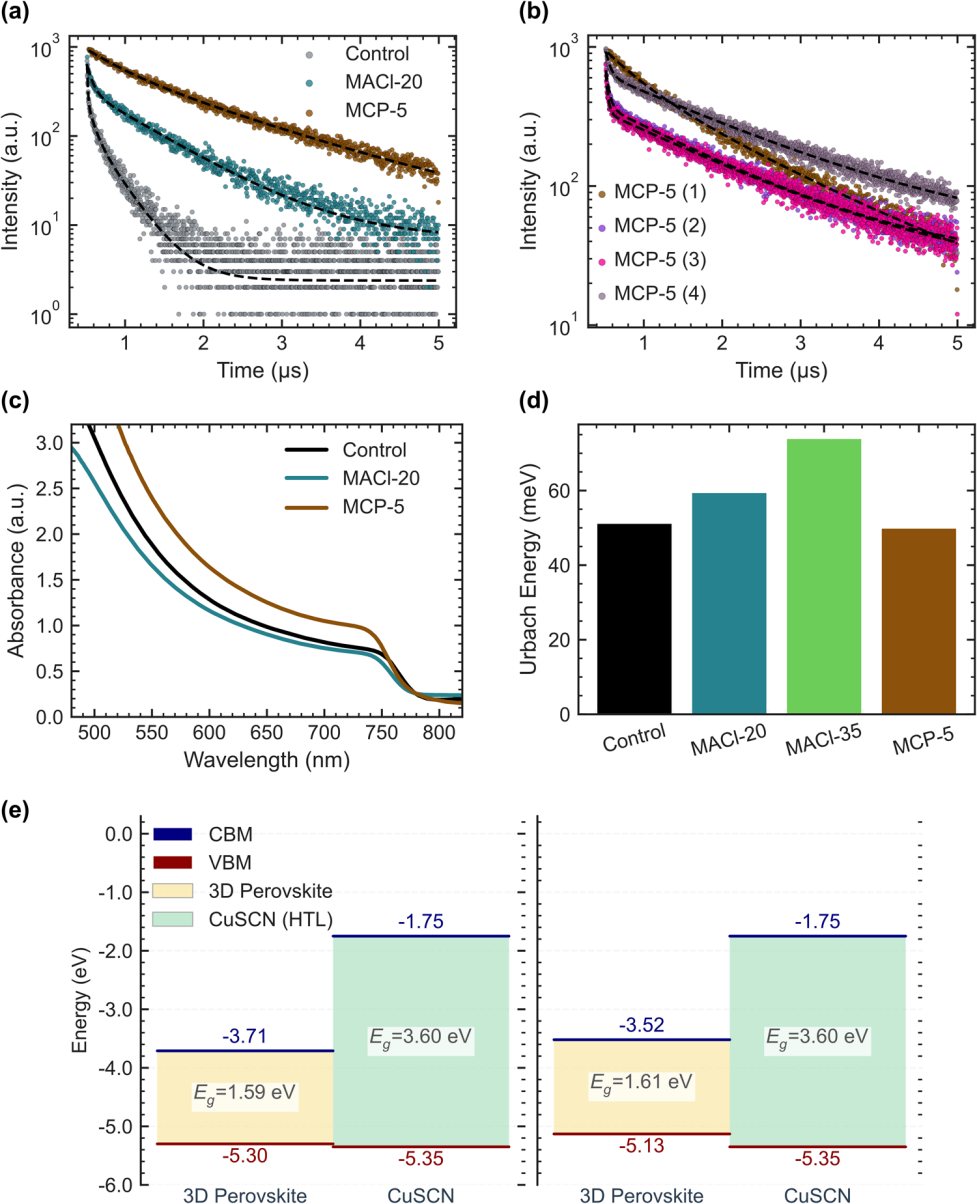
(a) TRPL spectra of control, MACl-20, and MCP-5 films, (b) TRPL spectra of multiple MCP-5 films, (c) UV–visible absorption spectra, (d) Urbach energy, and (e) energy-level alignment diagram as evaluated from PYSA spectra.

The MACl-20 film shows an intermediate lifetime of 820 ns (4.5-fold improvement over control), indicating that MACl can, in some instances, substantially suppress nonradiative recombination. However, this improvement in τ_avg_ does not resolve the reproducibility challenges identified earlier. As demonstrated in the PL statistical analysis (Figure 1c), the majority of MACl-20 samples exhibit PL quenching despite nominally identical processing conditions, with only a minority showing enhanced PL. The apparent contradiction—where a subset of MACl-processed samples exhibits long TRPL lifetimes, yet the majority shows reduced steady-state PL—supports a picture of strong spatial and sample-to-sample heterogeneity in defect passivation. In contrast, reproducibility testing on three additional MCP-5 films yields consistent lifetimes of 1.39, 1.53, and 1.66 μs (Figure 6b and Table S7), all significantly longer than the control. This narrow distribution, together with the uniformly enhanced PL intensity, stands in stark contrast to the bimodal PL response of MACl-processed films and demonstrates that MCP-5 enables reproducible, uniform defect passivation.

To assess the changes in optical bandgap (*E*_g_) and Urbach energy (*E*_u_) induced by the two chloride-based additives, ultraviolet–visible (UV–vis) absorption spectroscopy was performed. In Figures 4c and S16, the UV–vis absorption spectra of all films display the characteristic sharp absorption onset typical of lead halide perovskites, confirming the preservation of the perovskite phase upon additive incorporation. The optical bandgap, extracted by linear extrapolation of the Tauc plots (see Figure S17), systematically increases with MACl content from *E*_g_ = 1.59 eV (control) to 1.61 eV (MACl-20), 1.62 eV (MACl-35 and MACl-40), and 1.61 eV (MCP-5), indicating a progressive blue shift of the absorption edge consistent with chloride incorporation into the perovskite lattice and with the trends observed in the steady-state PL spectra.

Literature on triple-halide perovskites shows that MAPbCl_3_-type (MACl/PbCl_2_-derived) routes can act as more than a simple chloride donor: Xu et al. demonstrated that both MA^+^ and Cl^–^ originating from MAPbCl_3_ perovskite component may be incorporated into the perovskite lattice, yielding genuine mixed-A, mixed-halide perovskites with lattice contraction and bandgap widening as the MAPbCl_3_ content is increased.^48^ UV–vis absorption and steady-state PL spectra reveal a small blue shift of the band edge in both additive-treated films compared to the control, indicating a modest bandgap widening upon the addition of MACl and MCP-5. In MCP-5, this blue shift is accompanied by a slight shift of the main perovskite XRD reflections to higher 2θ, indicating lattice contraction. This explanation is consistent with lattice contraction, as expected when smaller species (Cl^–^ and/or MA^+^) partially incorporate into the perovskite lattice.^39,48^ By contrast, MACl-processed films exhibit bandgap widening together with the XRD peaks showing a net shift to smaller 2θ, corresponding to a lattice expansion when the peak doublets are analyzed in terms of their weighted-average position. This combination of bandgap widening with lattice expansion agrees with studies showing that tensile strain, Pb–X bond elongation, and octahedral distortions can increase the bandgap by reducing orbital overlap and modifying spin–orbit coupling, even when the lattice shows expansion.^72,73^ We therefore attribute the increase in bandgap in MACl-processed films primarily to distortion- and strain-mediated band-structure changes rather than to a simple Cl^–^ incorporation-driven lattice contraction.

Analysis of the sub-bandgap absorption tail shows that the Urbach energy increases from *E*_u_ = 50 meV (control) to 60 meV (MACl-20), whereas the MCP-5 film exhibits a slightly reduced *E*_u_ of 48 meV (Figure 6d); however, this reduction is relatively modest compared to the control. Figure S18 further reveals that *E*_u_ does not vary monotonically with increasing MACl concentration, reflecting the complex interplay between compositional modification and disorder in these films. The increase in *E*_u_ for the MACl-processed films indicates a broadening of the band-tail states and a higher density of sub-bandgap electronic states, pointing to enhanced energetic disorder and a greater population of shallow trap states.^74–76^

These optical trends, namely modest bandgap widening accompanied by increased energetic disorder, together with the microstrain extracted from the XRD peak broadening, provide a useful framework for interpreting the degraded optoelectronic characteristics and PL quenching observed in the MACl-processed films. In these samples, higher microstrain correlates with increased Urbach energy and stronger PL quenching. These findings corroborate the established mechanism that inhomogeneous lattice distortions promote the formation of nonradiative recombination centers near the band edges.^77^ In contrast, the MCP-5 film exhibits only a minor reduction in *E*_u_ (from 50 to 48 meV) yet shows a pronounced 7–8-fold enhancement in carrier lifetime and a microstrain level close to that of the control, indicating a partial decoupling of bulk energetic disorder from nonradiative recombination pathways. This disproportionate improvement suggests that the MCP additive more selectively passivates deep-level defects, such as undercoordinated Pb^2+^ sites or halide vacancies at grain boundaries, which act as the dominant Shockley-Read-Hall recombination centers, rather than significantly altering the overall band-tail width. Ni et al. have shown that deep trap states localized at interfaces can dominate carrier lifetime and device performance while contributing only weakly to the bulk-averaged sub-bandgap absorption tail, consistent with the limited change in *E*_u_ measured here.^78^ Accordingly, even though the global lattice/energetic disorder reflected in *E*_u_ is only marginally reduced in the MCP-5 film, the effective passivation of these killer deep traps leads, together with reduced microstrain, to a substantial suppression of nonradiative recombination. This result underscores that targeted passivation of grain boundaries and interfacial defects can yield order-of-magnitude improvements in optoelectronic quality without requiring a large change in the bulk Urbach energy.

Taken together, the XRD, PL, TRPL, and UV–Vis observations indicate that the bimodal PL response of MACl-processed films originates from MACl-induced halide segregation and structural heterogeneity rather than measurement artifacts. Steady state PL shows blue-shifted primary emission and a secondary emission around 810–815 nm, while XRD peak doublets, increased microstrain, and higher Urbach energies point to coexisting Br/Cl-rich and iodide-rich domains with distinct local strain and defect densities. This explanation is consistent with the Cl fraction at which triple-halide (I/Br/Cl) perovskites begin to phase-segregate decreases as the Br content is reduced, so that low-Br hosts such as FA_0.9_Cs_0.1_Pb­(I_0.9_Br_0.1_)_3_ (control) reach the phase boundary at comparatively modest Cl loadings.^39,48^ In contrast, MCP-5 films show single, symmetric XRD reflections, a single symmetric PL peak, and modest bandgap widening, consistent with a homogeneous triple-halide alloy and explaining the absence of bimodal PL and the superior reproducibility observed for MCP-5.

To study the impact of MCP on the energy-level alignment at the perovskite/CuSCN interface, we performed photoemission yield spectroscopy in air (PYSA) to extract the valence band maxima (VBM) of ITO, CuSCN, and the perovskite layers. PYSA yields VBMs of 4.71 eV for ITO and 5.35 eV for CuSCN, in good agreement with literature values for these materials (Figure S19). For the perovskite absorbers, the control film shows a VBM at 5.30 eV, whereas the MCP-5 film exhibits a slightly shallower VBM at 5.13 eV, indicating that the chloride additive subtly modified the electronic structure of the 3D perovskite surface. The energy band alignment of the full device, as shown in Figure 6e, was constructed using the PYSA results and the perovskite film bandgap determined from the absorption spectra. Relative to CuSCN, this corresponds to a nearly resonant valence band offset of ∼0.05 eV for the control perovskite and a larger offset of ∼0.22 eV for MCP-5. These findings imply a higher nominal energy barrier for hole transfer in the MCP-5-based devices than in the control. The shift in VBM observed in our MCP-5-alloyed films is consistent with theoretical investigations of perovskite systems, which highlight the sensitivity of the electronic band structure to the chemical environment and site-specific orbital contributions.^7,79^

### Device Performance

To evaluate the impact of chloride-based additives on photovoltaic performance, single-junction PSCs were fabricated using an n-i-p architecture: glass/ITO/perovskite/OAI/CuSCN/Au (Figure 7a). Figure 7b presents the current density–voltage (*J*–*V*) curves of the champion devices for the control, MACl-20, and MCP-5 modifications. The control device achieved a PCE of 10.06%, with an open-circuit voltage (*V*_OC_) of 1.00 V, a short-circuit current density (*J*_SC_) of 16.67 mA/cm^2^, and a fill factor (FF) of 60.32%. The MACl-20-based device yielded a similar PCE of 10.05% (*V*_OC_ = 1.00 V, *J*_SC_ = 17.23 mA/cm^2^, FF = 58.31%), showing a slight enhancement in photocurrent but no net efficiency gain. In contrast, the MCP-5-based device provided a higher PCE of 12.03%, driven by simultaneous modest increases in *V*_OC_ to 1.03 V and *J*_SC_ to 19.63 mA/cm^2^, while maintaining a comparable FF of 59.51%. The active area of each device was 0.2 cm^2^, and we regard these efficiencies as modest but representative for comparing the MACl-20 and MCP-5 additive-processing routes under the conditions used in this work.

**Figure 7:**
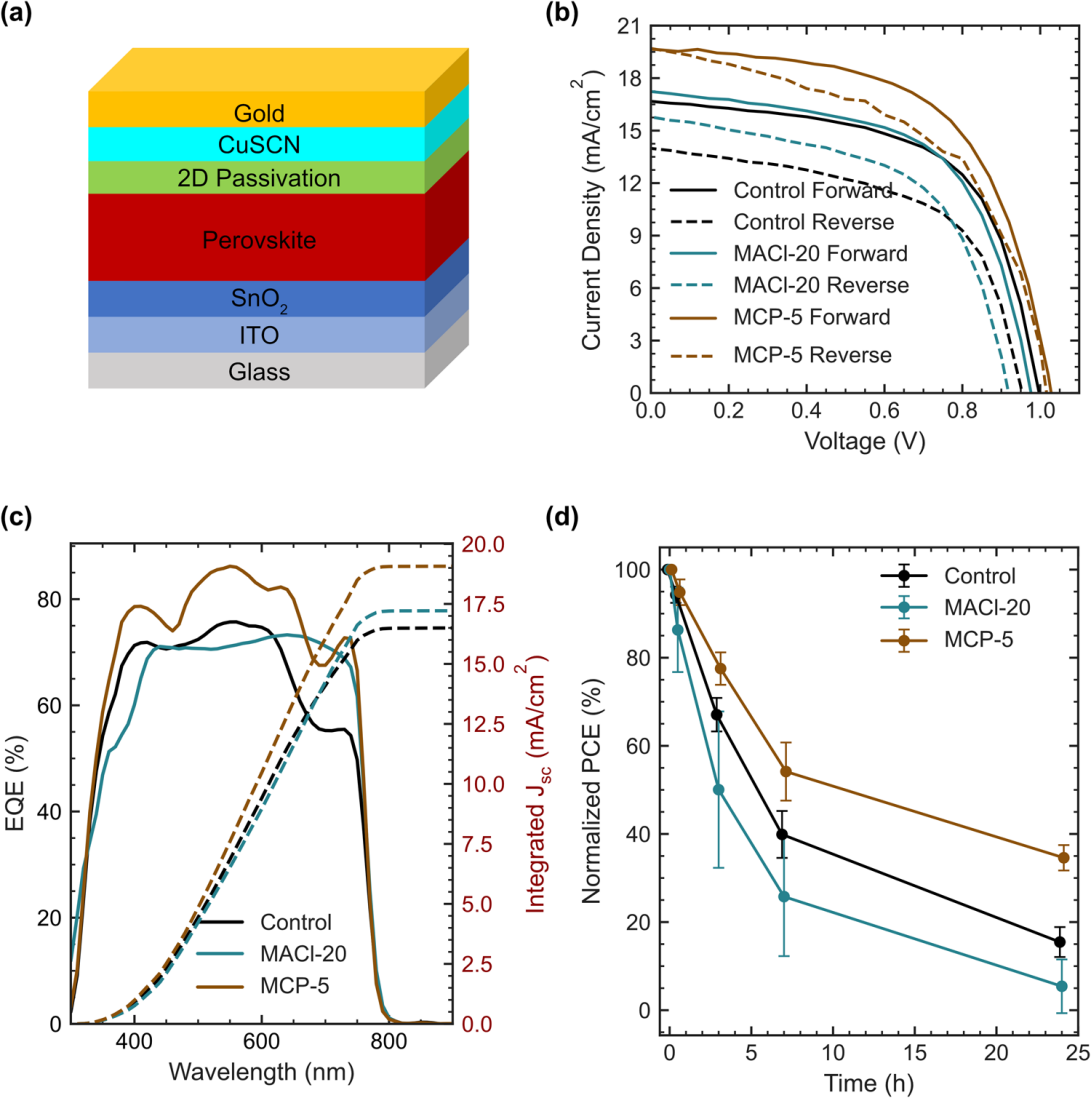
(a) PSC structure, (b) *J*–*V* Curves, (c) EQE, and (d) stability of PSCs with and without chloride-based additives.

The modest *V*_OC_ increase of 30 mV for MCP-5 (1.00 to 1.03 V) is noticeably smaller than the large relative enhancements observed in PL intensity and TRPL lifetime, which indicate a substantial reduction in bulk trap-assisted nonradiative recombination in the perovskite absorber. In light of the PYSA results, this apparent mismatch can be rationalized by the nonideal perovskite/HTL energetics: the MCP-5 film exhibits a relatively shallow VBM at 5.13 eV, while CuSCN has a deep VBM at 5.35 eV, leading to a valence band offset of ∼0.22 eV at the perovskite/CuSCN side. Together with residual nonradiative recombination at the low-temperature SnO_2_/perovskite and perovskite/CuSCN interfaces, this valence band offset implies that *V*_OC_ in our architecture remains limited predominantly by interfacial losses and nonideal energy-level alignment, even though nonradiative recombination in the MCP-5 film is strongly suppressed.

The observed *J*_SC_ values (16.67–19.63 mA/cm^2^) indicate nonoptimized charge-transport layer architectures at both interfaces. At the electron transport layer (ETL), low-temperature processed SnO_2_ (150 °C, 30 min) can exhibit oxygen vacancy defect concentrations that trap electrons and accelerate perovskite degradation,^80^ while the absence of interfacial passivation layers (KCl, SnF_2_) eliminates performance enhancements. At the HTL, solution-processed CuSCN can introduce losses through (i) solvent-induced perovskite surface degradation creating trap states,^81^ and (ii) interfacial incompatibility with 2D passivation, reducing CuSCN wettability, leading to nonuniform HTL coverage and morphological defects.^82^ We attribute these cumulative interface losses to the *J*_SC_ deficit relative to optimized devices. Since this investigation focused on chloride additive optimization rather than transport-layer engineering, interface parameters remained nonoptimized to isolate additive-induced performance modifications. The systematic *J*_SC_ enhancement from 16.67 to 19.63 mA/cm^2^ (∼18% improvement) with MCP-5 modification demonstrates effective improvement in intrinsic perovskite quality. Future work optimizing device architecture would substantially increase absolute device performance toward theoretical limits while preserving the mechanistic material insights demonstrated herein.

To verify reproducibility, statistical data were collected from 10 independent devices per condition (Figure S20 and Table 1). The MCP-5-based devices demonstrated superior consistency and average performance, yielding a PCE of 10.60 ± 0.97%, significantly higher than the control average of 8.43 ± 0.90%. In contrast, the MACl-20-based devices exhibited a lower average PCE of 5.88 ± 2.20% with a notably larger standard deviation, indicating poor process reproducibility. This clear divergence between MACl-20 and MCP-5 is consistent with the optoelectronic characterization: MCP-5 yields uniformly enhanced PL intensity and extended TRPL lifetimes, whereas MACl-20 produces bimodal PL responses and structural heterogeneity, leading to unstable device performance across nominally identical processing conditions.

**Table 1:** Photovoltaic Parameters of FA_0.9_Cs_0.1_Pb­(I_0.9_Br_0.1_)_3_ PSCs Based on Different Additives: Control, MACl-20, and MCP-5^a^

Sample	*J*_SC_ (mA/cm^2^)	*V*_OC_ (V)	FF (%)	PCE (%)
Control	17.19 ± 1.66 (16.67)	0.94 ± 0.06 (1)	52.51 ± 5.61 (60.32)	8.43 ± 0.90 (10.06)
MACl-20	15.70 ± 3.53 (17.23)	0.86 ± 0.15 (1)	43.24 ± 9.57 (58.31)	5.88 ± 2.20 (10.05)
MCP-5	18.96 ± 1.03 (19.63)	0.99 ± 0.05 (1.03)	56.95 ± 2.56 (59.61)	10.63 ± 1.01 (12.03)

a Results in parentheses are the champion results.

Figure 7c shows the External Quantum Efficiency (EQE) spectra along with the integrated *J*_SC_ for control, MACl-20, and MCP-5-based PSCs. The control device exhibited a moderate photoresponse, with a pronounced decline in the long-wavelength region (600–750 nm), suggesting limited carrier diffusion lengths in the bulk film. In contrast, the MCP-5-based device demonstrated a significantly enhanced broadband response, maintaining an EQE exceeding 80% across the entire visible spectrum. This improvement indicates that the addition of MCP not only improves film quality but also suppresses recombination channels. While the MACl-20-based device shows improved response in the long-wavelength region compared to the control, it suffered from a distinct loss in the short-wavelength region (300–500 nm). This blue loss is attributed to increased surface recombination or parasitic absorption at the front interface. The integrated *J*_SC_ calculated from EQE spectra were 16.50, 17.21, and 19.07 mA/cm^2^ for the control, MACl-20, and MCP-5-based devices, respectively. Notably, the integrated *J*_SC_ aligns well with the values obtained from the *J*–*V* measurements, confirming the reliability of the PV performance enhancement.

To assess the operational stability under humid conditions, unencapsulated devices were stored under ambient air at a high relative humidity (RH) of 70–75%. Figure 7d shows the normalized PCE as a function of storage time over 24 h. Under these harsh conditions, the MCP-5-based devices retained ∼36.7% of their initial PCE after 24 h, compared to only ∼14.3% retention for the control devices and a rapid decay to ∼0.2% for MACl-20-treated devices. For each condition, three devices were monitored, and Figure 7d shows the mean normalized PCE, with error bars indicating the standard deviation across the three devices. Within this short-term, unencapsulated humidity test, MCP-5 thus affords improved PCE retention relative to both the control and MACl-20 routes, whereas MACl-containing devices are particularly susceptible to rapid performance loss under humid exposure, consistent with the known instability of residual MACl and the poor reproducibility observed in their device statistics. We note that these measurements reflect only short-term humidity tolerance for three devices per condition under the specific conditions used here and do not constitute a full long-term stability assessment.

Our results demonstrate the MCP additive strategy for a representative FA_0.9_Cs_0.1_Pb­(I_0.9_Br_0.1_)_3_ composition, which exhibits a moderately increased bandgap relative to pure iodide analogs. Together with previous reports by Xu et al. and co-workers,^48^ who used MCP to realize triple-halide and other mixed-cation/mixed-halide perovskites with improved bandgap control and suppressed phase segregation, this suggests that a nonvolatile MCP source is a broadly promising route to homogenize chloride incorporation in related wide-bandgap systems. Nonetheless, systematic studies across additional A-site and halide combinations will be required to fully establish the scope and limits of this approach.

## Conclusion

In summary, we systematically investigated the reproducibility of chloride-based additive engineering in FA_0.9_Cs_0.1_Pb­(I_0.9_Br_0.1_)_3_ perovskite solar cells. Although MACl is widely used, it induces a distinctly bimodal photoluminescence (PL) response, with most nominally identical films exhibiting PL quenching rather than enhancement. By combining XRD, line-profile analysis, atomic force microscopy (AFM), and time-resolved photoluminescence (TRPL), we attribute this variability to compositional and structural heterogeneity, including halide-rich phase coexistence and nonuniform microstrain distributions that promote trap-assisted nonradiative recombination. In contrast, MCP functions as a robust additive that reliably produces PL enhancement across all samples, increasing the carrier lifetime from 182 to 1663 ns, consistent with strongly suppressed nonradiative losses. PSCs fabricated with MCP and CuSCN as a low-cost hole-transport layer achieve a PCE of 12% with an open-circuit voltage (*V*_OC_) of 1.03 V—a ∼20% relative improvement over MACl-processed devices—and exhibit significantly reduced device-to-device variation compared to MACl-processed devices alongside enhanced short-term humidity stability of unencapsulated devices. Collectively, these findings identify process sensitivity as a critical yet underrecognized limitation of MACl and establish rational additive selection––specifically the use of MCP–– as a viable route toward reproducible perovskite photovoltaics that can be extended to other mixed-cation/mixed-halide compositions.

## Supplementary Material



## Notes

Additional data in the form of bootstrap confidence intervals (95% Cl, 10,000 resamples) for mean PL peak intensity, quantile-percentile plot of PL intensity for each condition, PL spectra, XRD Patterns, W–H Plots, H–W Plots, AFM images, grain-size distribution, PSD profiles, Tauc plots, Urbach energy analysis plots and device statistics (PDF)
