# Supplementary material for: Stoichiometric MACl/PbCl2 Additive Engineering for Improved Reproducibility and Carrier Lifetime in FA/Cs Mixed-Halide Perovskite Solar Cells
Source: ACS Omega. 2026 Jul 29;11(31):45815–31. doi: 10.1021/acsomega.6c01439 (PMC13470813; doi:10.1021/acsomega.6c01439)
Supplement: Supplementary file 1 [file ao6c01439_si_001.pdf]

# Supporting Information

# Stoichiometric MACl/$PbCl_2$ Additive Engineering for Improved Reproducibility and Carrier Lifetime in FA/Cs Mixed-Halide Perovskite Solar Cells

*Vishal Viswakarman* [a,c], *Miguel García Rocha* [a,b,*], *Johann Bouclé* [c,*]

[a]Program on Nanoscience and Nanotechnology, Centro de Investigación y de Estudios Avanzados del Instituto Politécnico Nacional (CINVESTAV-IPN), Av. Instituto Politécnico Nacional 208, Col. San Pedro Zacatenco, Ciudad de México, Código Postal 07360, Mexico

[b]Department of Physics, Centro de Investigación y de Estudios Avanzados del Instituto Politécnico Nacional (CINVESTAV-IPN), Av. Instituto Politécnico Nacional 208, Col. San Pedro Zacatenco, Ciudad de México, Código Postal 07360, Mexico

[c]Univ. Limoges, CNRS, XLIM, UMR 7252, F-87000 Limoges, France

*Corresponding author: johann.boucle@unilim.fr, mgarciar@cinvestav.mx

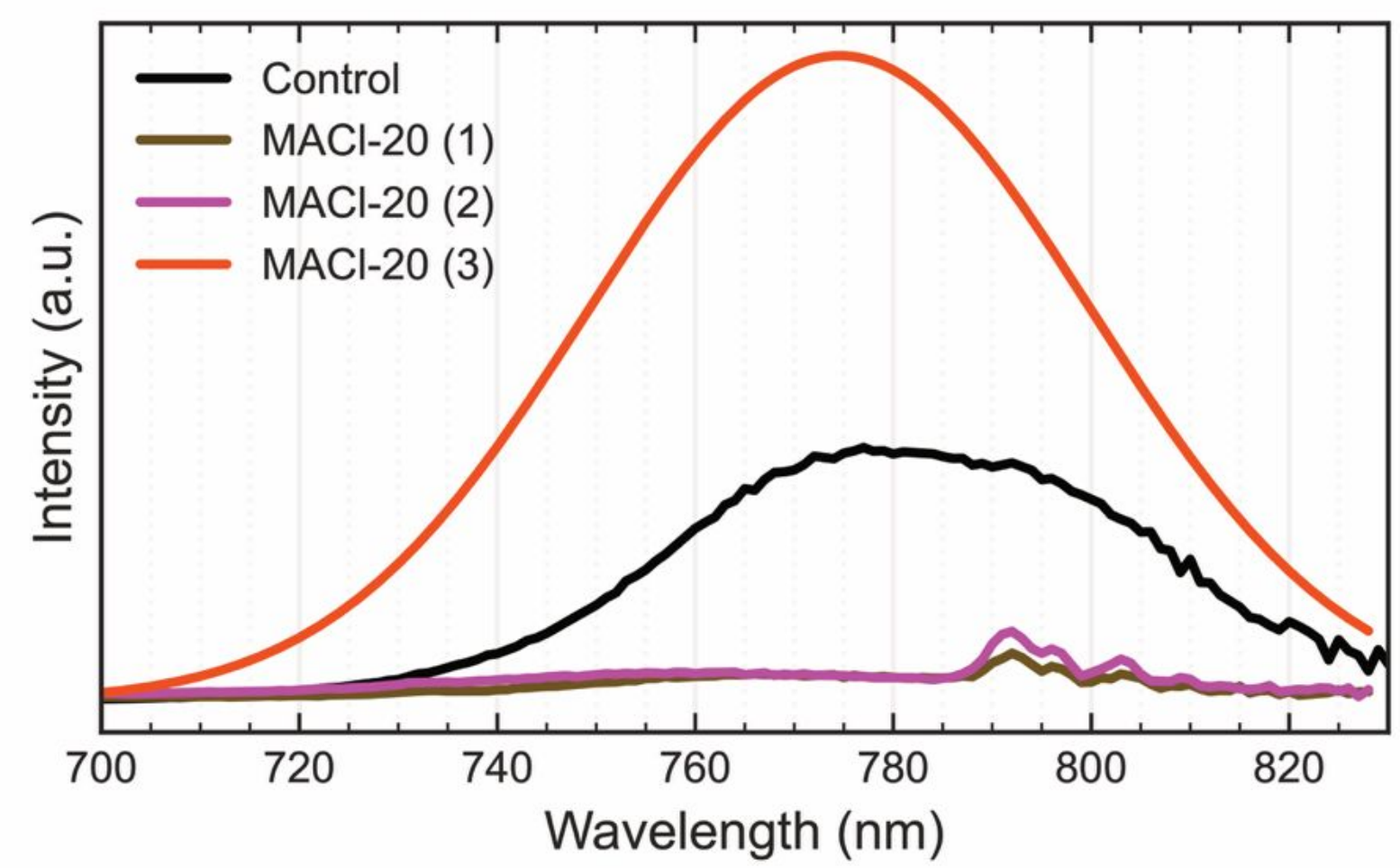


**Figure S1:** Steady-state PL spectra of control and MACl-20-treated $FA_{0.9}Cs_{0.1}Pb(I_{0.9}Br_{0.1})_3$ perovskite films processed inside a $N_2$ glovebox. The three MACl-20 films show strong sample-to-sample variation in PL intensity and spectral shape, indicating that the heterogeneous PL response of MACl-20 is not eliminated by glovebox processing. For each film, the PL intensity has been normalized by the absorbance at the excitation wavelength to account for differences in optical density.

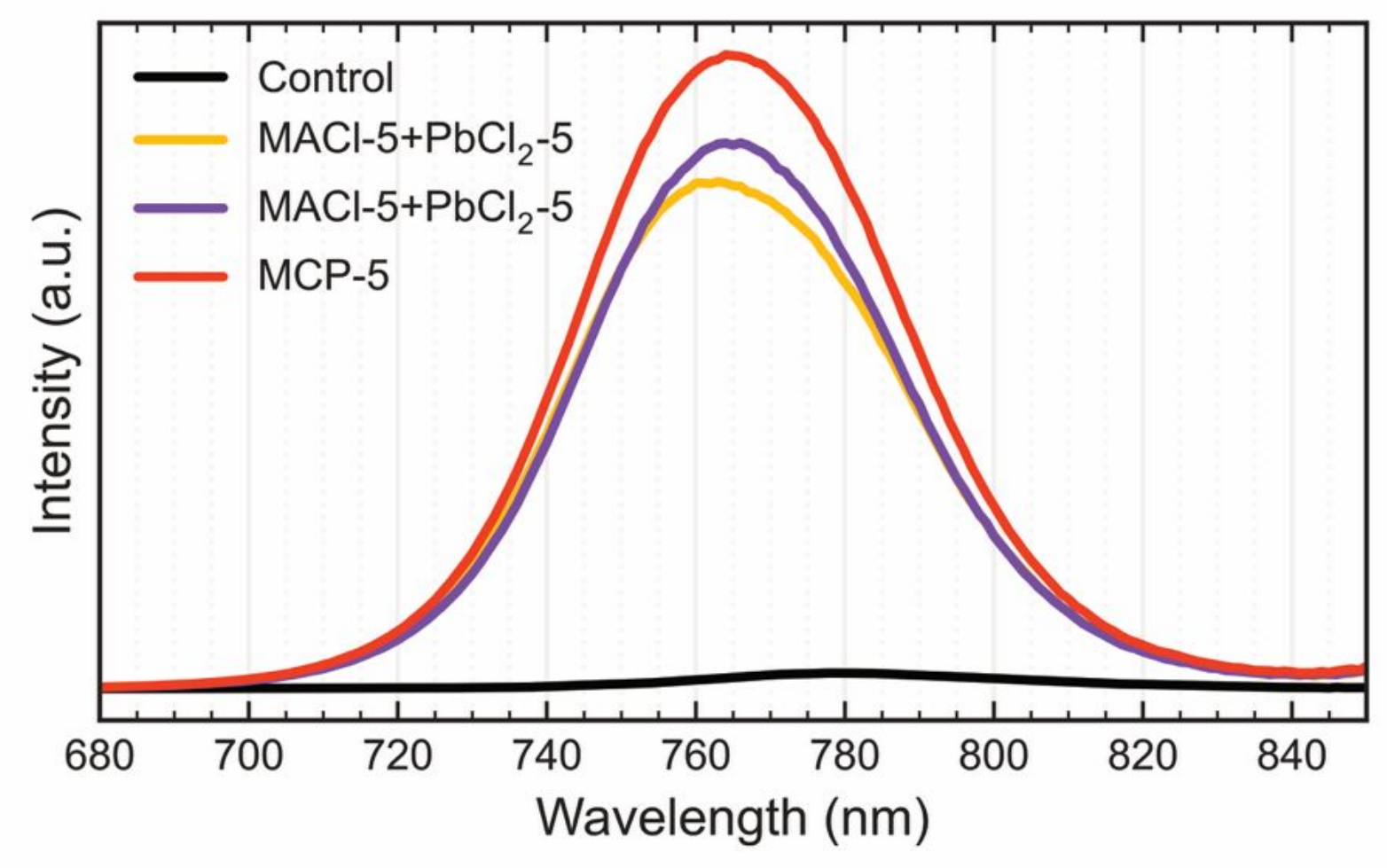


**Figure S2:** Steady-state PL spectra of control, directly mixed MACl-5+$PbCl_2$-5, and MCP-5-derived perovskite films. The similar PL response of the directly mixed MACl+$PbCl_2$ and MCP-derived films suggests that the observed optoelectronic effect is associated with the stoichiometric MACl/$PbCl_2$-derived chloride additive formulation, rather than requiring a distinct residual crystalline MCP phase. For each film, the PL intensity has been normalized by the absorbance at the excitation wavelength to account for differences in optical density.

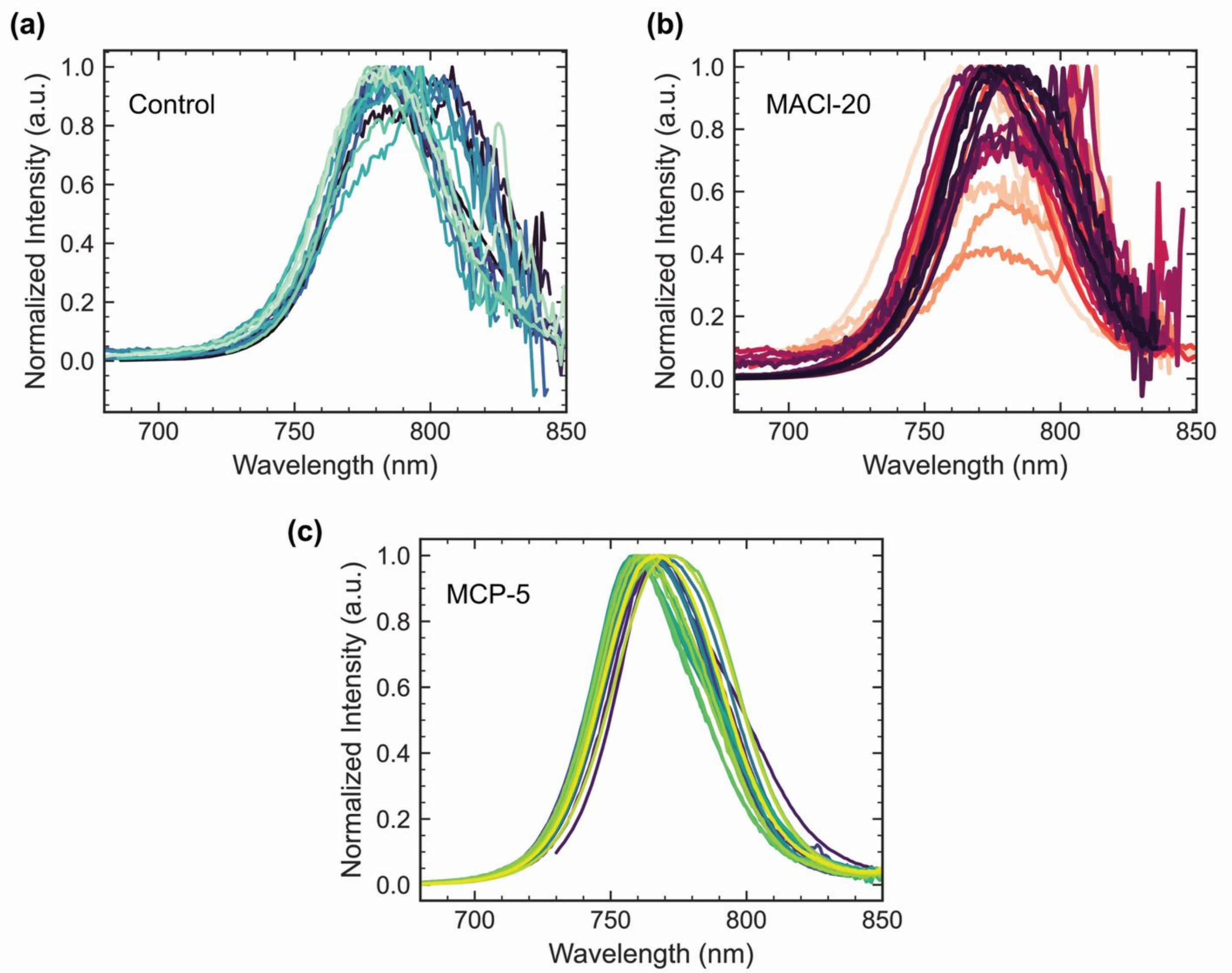


**Figure S3.** Normalized steady-state PL spectra of (a) Control, (b) MACl-20, and (c) MCP-5 $FA_{0.9}Cs_{0.1}Pb(I_{0.9}Br_{0.1})_3$ perovskite films used in the statistical analysis. Each spectrum is normalized to its own maximum intensity ($I/I_{max}$) to highlight sample-to-sample variations in spectral shape and peak position across independently fabricated films, without additional absorbance normalization.

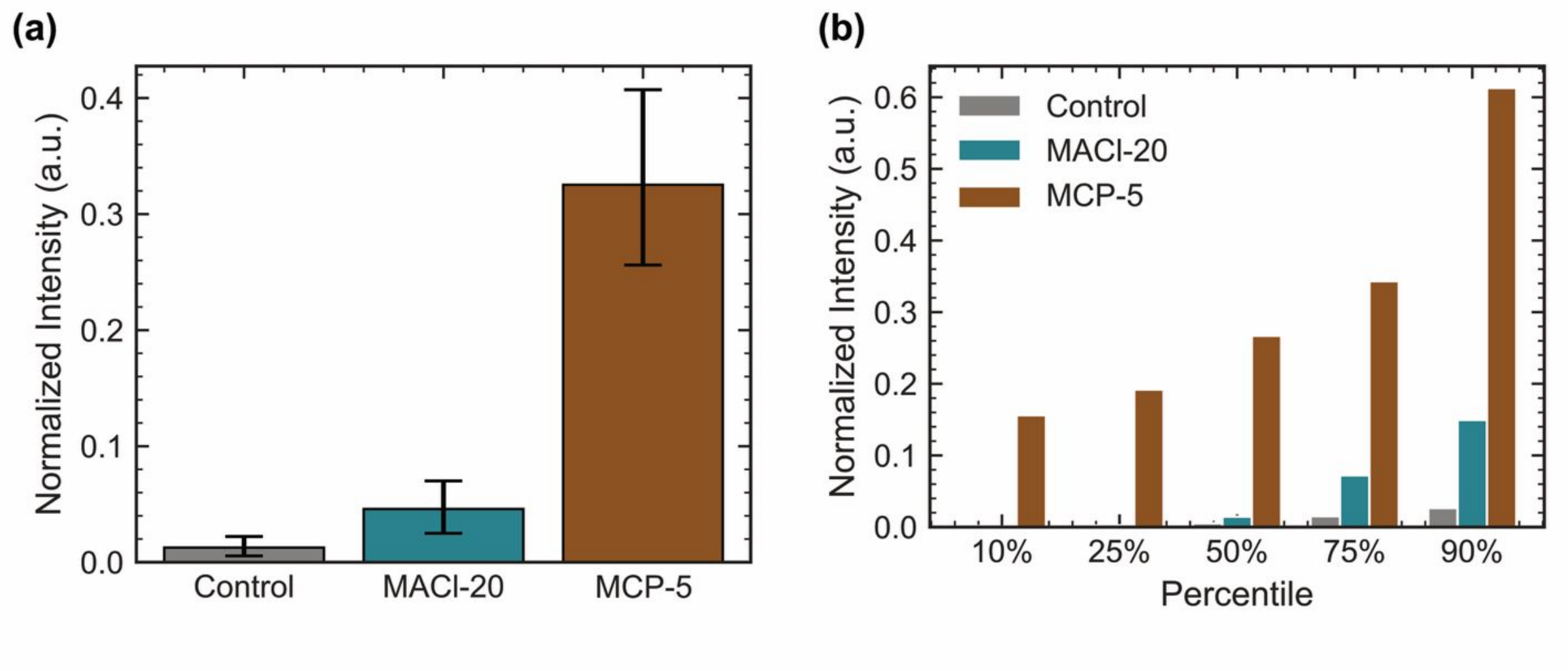


**Figure S4:** Statistical Analysis of raw PL peak intensity distributions. (a) Bootstrap confidence intervals (95% Cl, 10,000 resamples) for mean raw PL peak intensity across control, MACl-20, and MCP-5 films (n=25 films per condition). (b) Quantile plot showing raw PL peak intensity at the 10th, 25th, 50th, 75th, and 90th percentiles for each condition.

**Table S1:** Non-parametric pairwise comparisons using Mann-Whitney U tests

| Comparison | Mann-Whitney U | p-value | Effect Size (r) | Vargha-Delaney $A_{12}$ | Interpretation |
|---|---|---|---|---|---|
| **Control vs MACl-20** | 187.00 | 0.147 | 0.216 (small) | 0.371 (small) | No significant difference |
| **Control vs MCP-5** | 0.00 | $1.92 \times 10^{-8}$ | 0.839 (large) | 0.000 (large) | MCP-5 dominant |
| **MACl-20 vs MCP-5** | 20.00 | $1.75 \times 10^{-9}$ | 0.813 (large) | 0.026 (large) | MCP-5 dominant |

**Table S2:** Bootstrap analysis of mean PL intensity

| Condition | Mean | 95% Cl Lower | 95% Cl Upper |
|---|---|---|---|
| **Control** | $1.26 \times 10^{-2}$ | $5.59 \times 10^{-3}$ | $2.25 \times 10^{-2}$ |
| **MACl-20** | $4.58 \times 10^{-2}$ | $2.44 \times 10^{-2}$ | $7.02 \times 10^{-2}$ |
| **MCP-5** | $3.25 \times 10^{-1}$ | $2.55 \times 10^{-1}$ | $4.08 \times 10^{-1}$ |

**Table S3:** Bootstrap difference tests between conditions

| Comparison | Mean Difference | p-value | Significance |
|---|---|---|---|
| **Control vs MACl-20** | $-3.33 \times 10^{-2}$ | 0.026 | * |
| **Control vs MCP-5** | $-3.13 \times 10^{-1}$ | <0.001 | *** |
| **MACl-20 vs MCP-5** | $-2.79 \times 10^{-1}$ | <0.001 | *** |

***Significance levels:*** * $p < 0.05$, **$p < 0.01$, ***$p < 0.001$

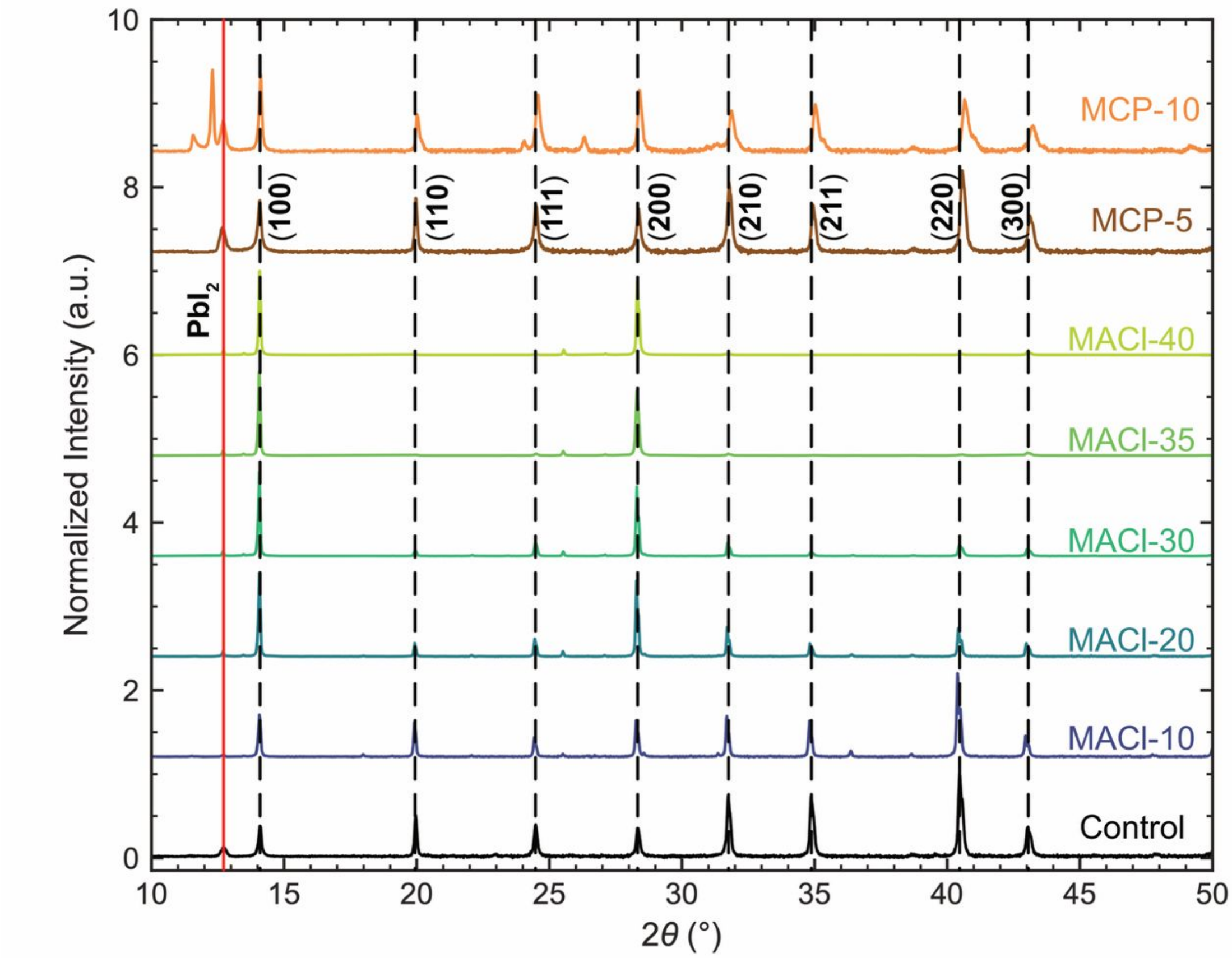


**Figure S5:** Normalized full-range XRD patterns of $FA_{0.9}Cs_{0.1}Pb(I_{0.9}Br_{0.1})_3$ perovskite films as a function of additive concentration. The dashed black lines mark the indexed perovskite reflections, while the red vertical line indicates the $PbI_2$-related reflection near 12.7°.

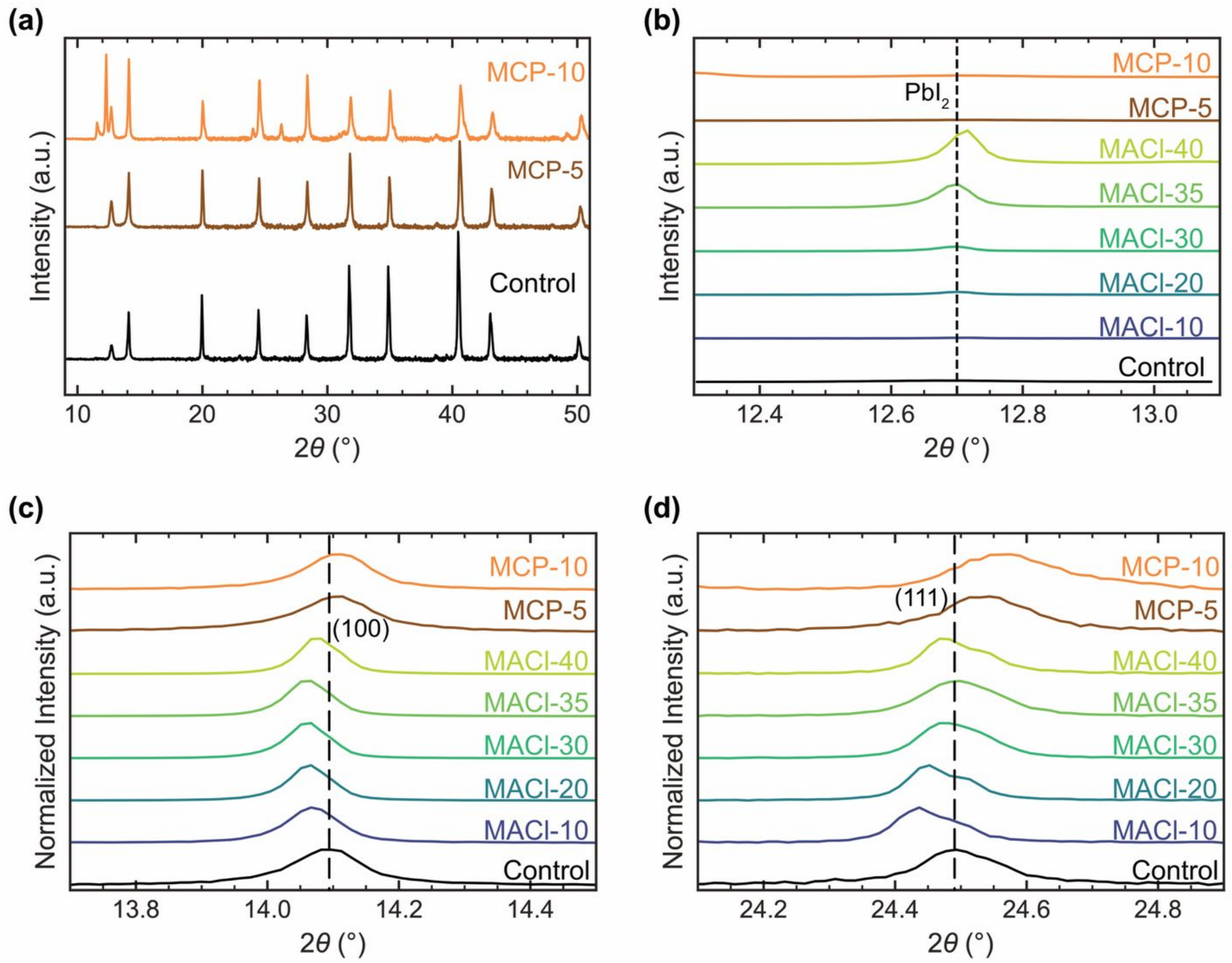


**Figure S6.** (a) Full XRD patterns of selected control and MCP-derived films. (b) Expanded low-angle region showing the $PbI_2$-related reflection near 12.7°. (c) Expanded primary perovskite peak region near 14.1°, showing the evolution of the (100) reflection and (d) XRD patterns tracking the evolution of (111) peak.

**Table S4:** Instrumental Broadening Analysis

| Sample | Peak | $\beta_{obs}$ (º) | $\beta_{inst}$ (º) | $\beta_{sample}$ (º) | $\beta_{sample}/\beta_{inst}$ |
|---|---|---|---|---|---|
| Control | (100) | 0.1391 | 0.055 | 0.1278 | 2.324 |
| | (200) | 0.1634 | 0.055 | 0.1539 | 2.798 |
| | (300) | 0.2122 | 0.046 | 0.2072 | 4.504 |
| MACl-10 | (100) | 0.0785 | 0.055 | 0.0561 | 1.02 |
| | (200) | 0.1072 | 0.055 | 0.0921 | 1.674 |
| | (300) | 0.1479 | 0.046 | 0.1407 | 3.059 |
| MACl-20 | (100) | 0.0637 | 0.055 | 0.0321 | 0.584 |
| | (200) | 0.0919 | 0.055 | 0.0737 | 1.34 |
| | (300) | 0.1426 | 0.046 | 0.1350 | 2.934 |
| MACl-30 | (100) | 0.063 | 0.055 | 0.0307 | 0.558 |
| | (200) | 0.0911 | 0.055 | 0.0727 | 1.322 |
| | (300) | 0.1619 | 0.046 | 0.1553 | 3.376 |
| MACl-35 | (100) | 0.0653 | 0.055 | 0.0351 | 0.638 |
| | (200) | 0.1057 | 0.055 | 0.0903 | 1.641 |
| | (300) | 0.1810 | 0.046 | 0.1751 | 3.806 |
| MACl-40 | (100) | 0.0612 | 0.055 | 0.0267 | 0.485 |
| | (200) | 0.0973 | 0.055 | 0.0803 | 1.46 |
| | (300) | 0.1506 | 0.046 | 0.1435 | 3.119 |
| MCP-5 | (100) | 0.1105 | 0.055 | 0.0958 | 1.741 |
| | (200) | 0.1400 | 0.055 | 0.1287 | 2.34 |

| | | | | | |
|---|---|---|---|---|---|
| | (300) | 0.1846 | 0.046 | 0.1788 | 3.886 |
| MCP-10 | (100) | 0.1029 | 0.055 | 0.087 | 1.581 |
| | (200) | 0.1481 | 0.055 | 0.1375 | 2.55 |
| | (300) | 0.2823 | 0.046 | 0.2786 | 6.056 |

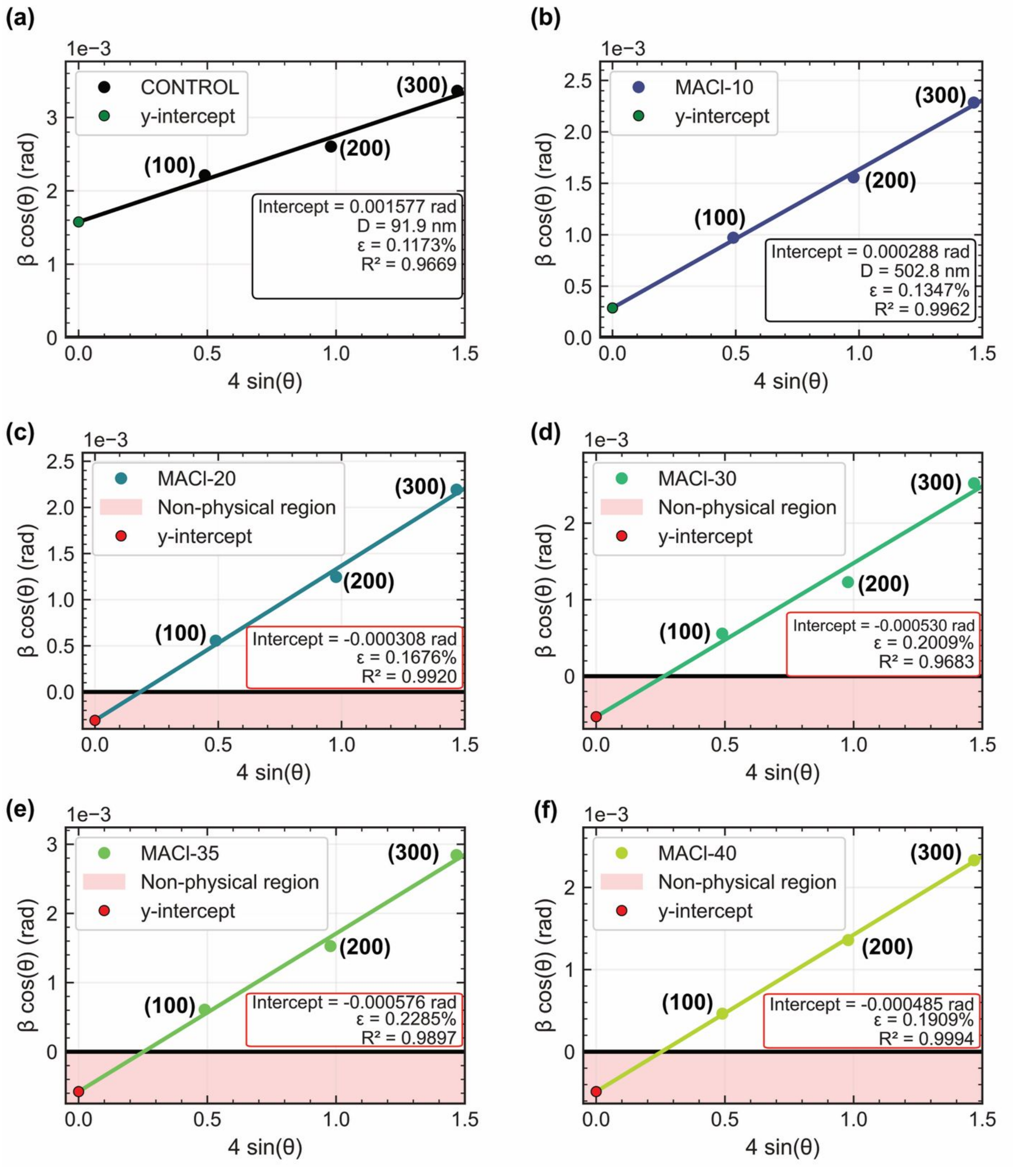


**Figure S7.** W-H plots from the XRD patterns of (a) Control, (b) MACl-10, (c) MACl-20, (d) MACl-30, (e) MACl-35, and (f) MACl-40 films.

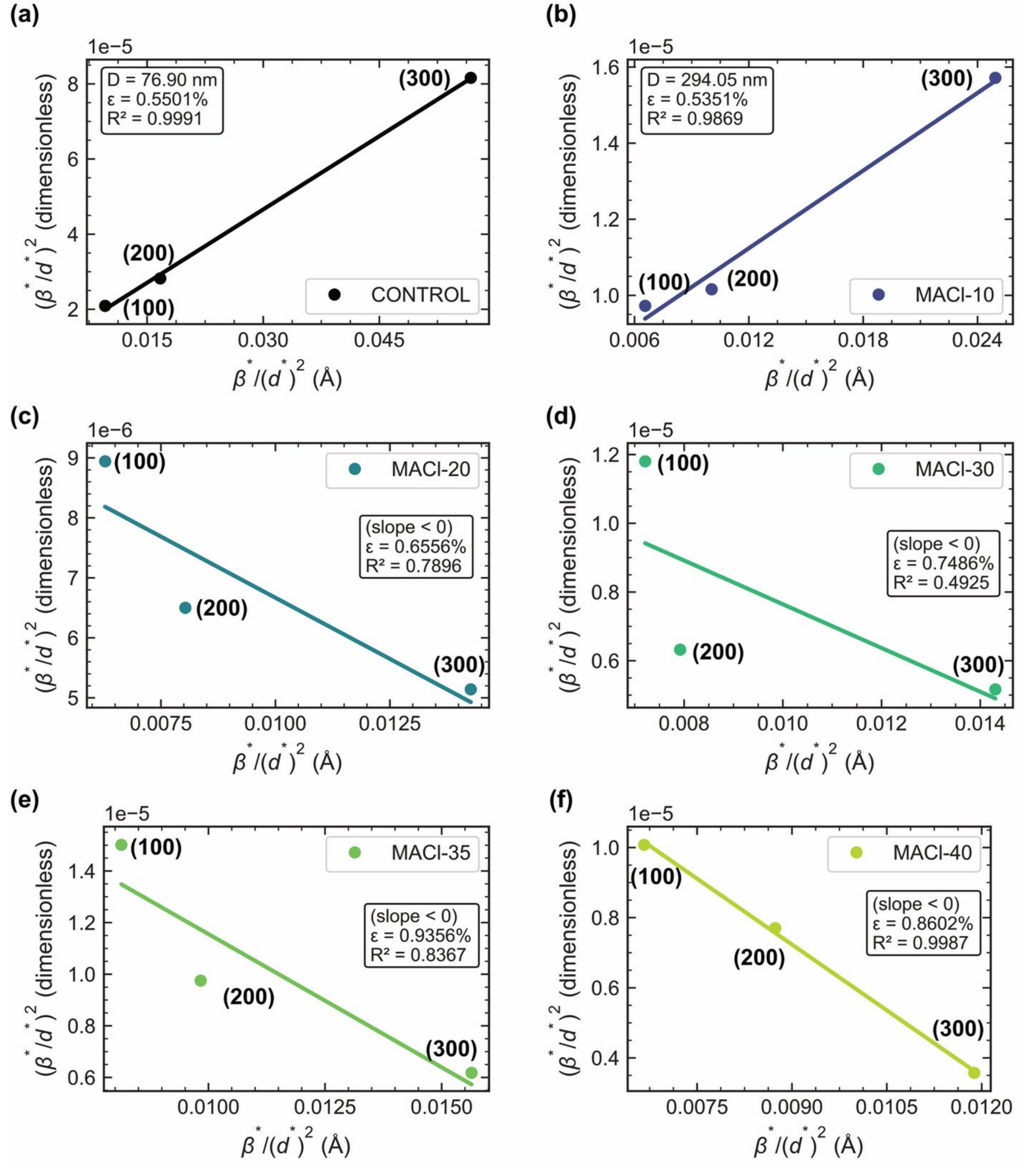


**Figure S8.** H-W plots from the XRD patterns of (a) Control, (b) MACl-10, (c) MACl-20, (d) MACl-30, (e) MACl-35, and (f) MACl-40 films.

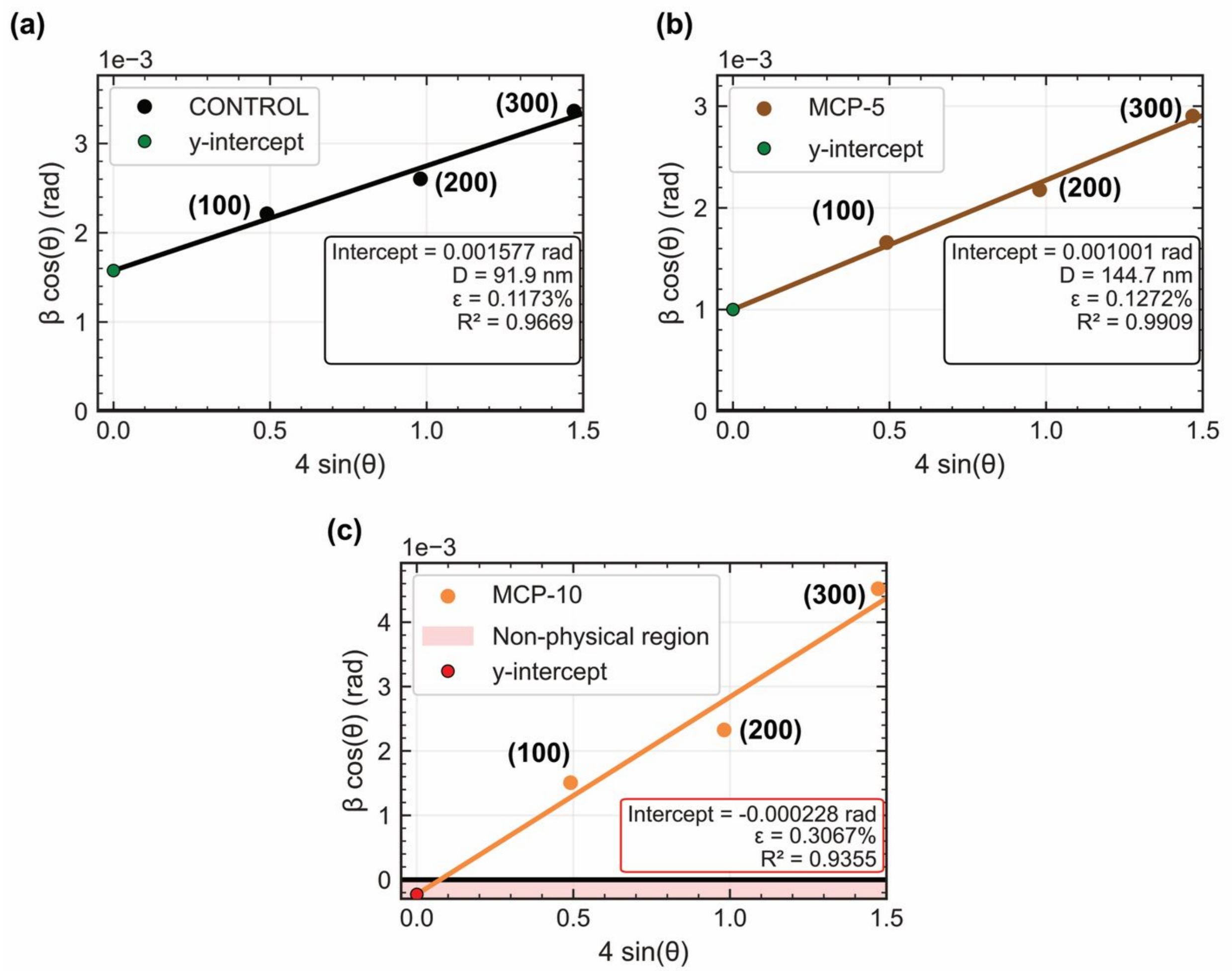


**Figure S9:** W–H plots of (a) Control, (b) MCP-5, and (c) MCP-10 perovskite films. Linear fits of β.cos(θ) as a function of 4.sin(θ) were used to estimate crystallite size and microstrain from XRD peak broadening.

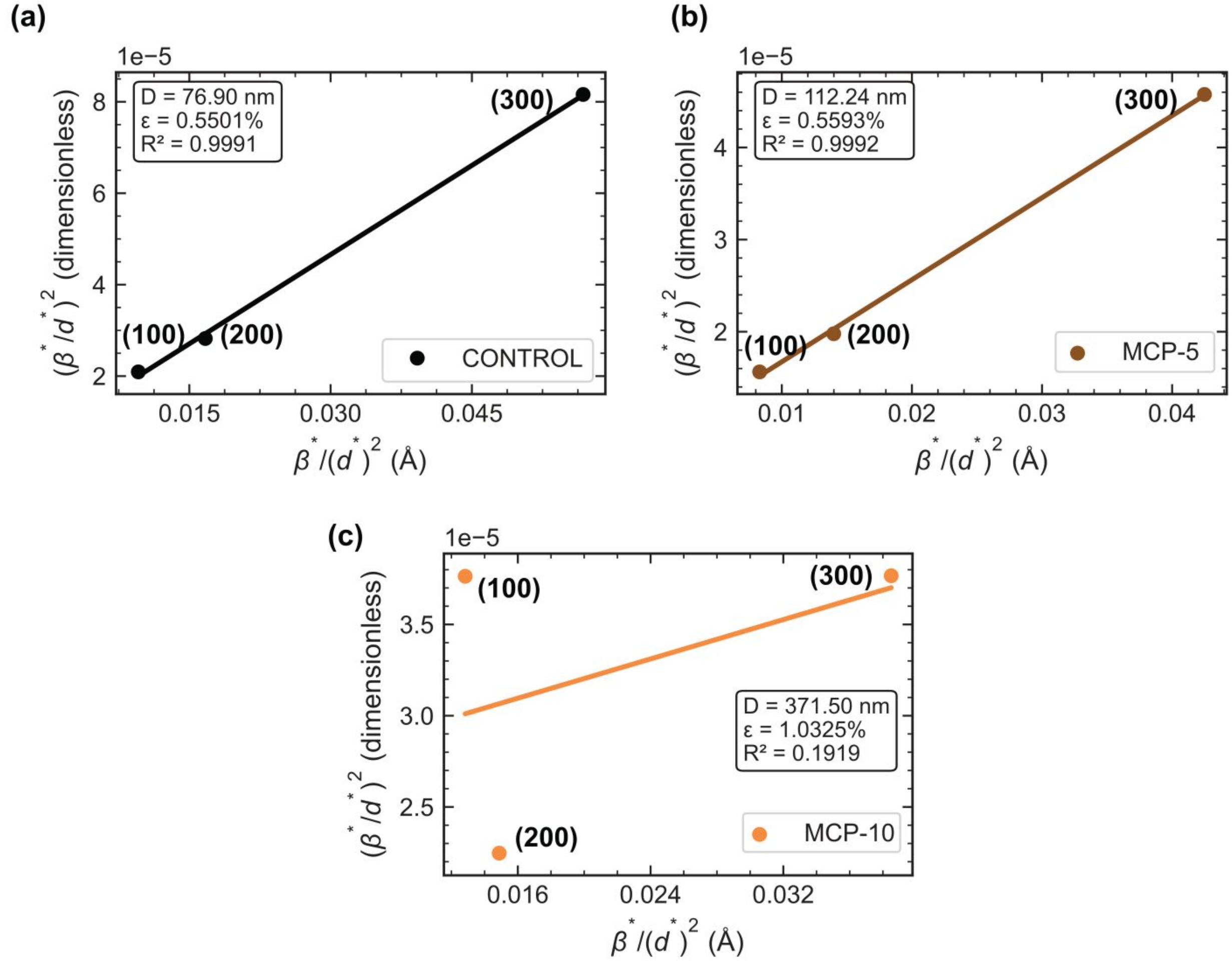


**Figure S10:** H–W plots of (a) control, (b) MCP-5, and (c) MCP-10 perovskite films. The analysis was performed using the indexed perovskite reflections, and the linear fits were used to estimate crystallite size, D, and microstrain, ε, from XRD peak broadening.

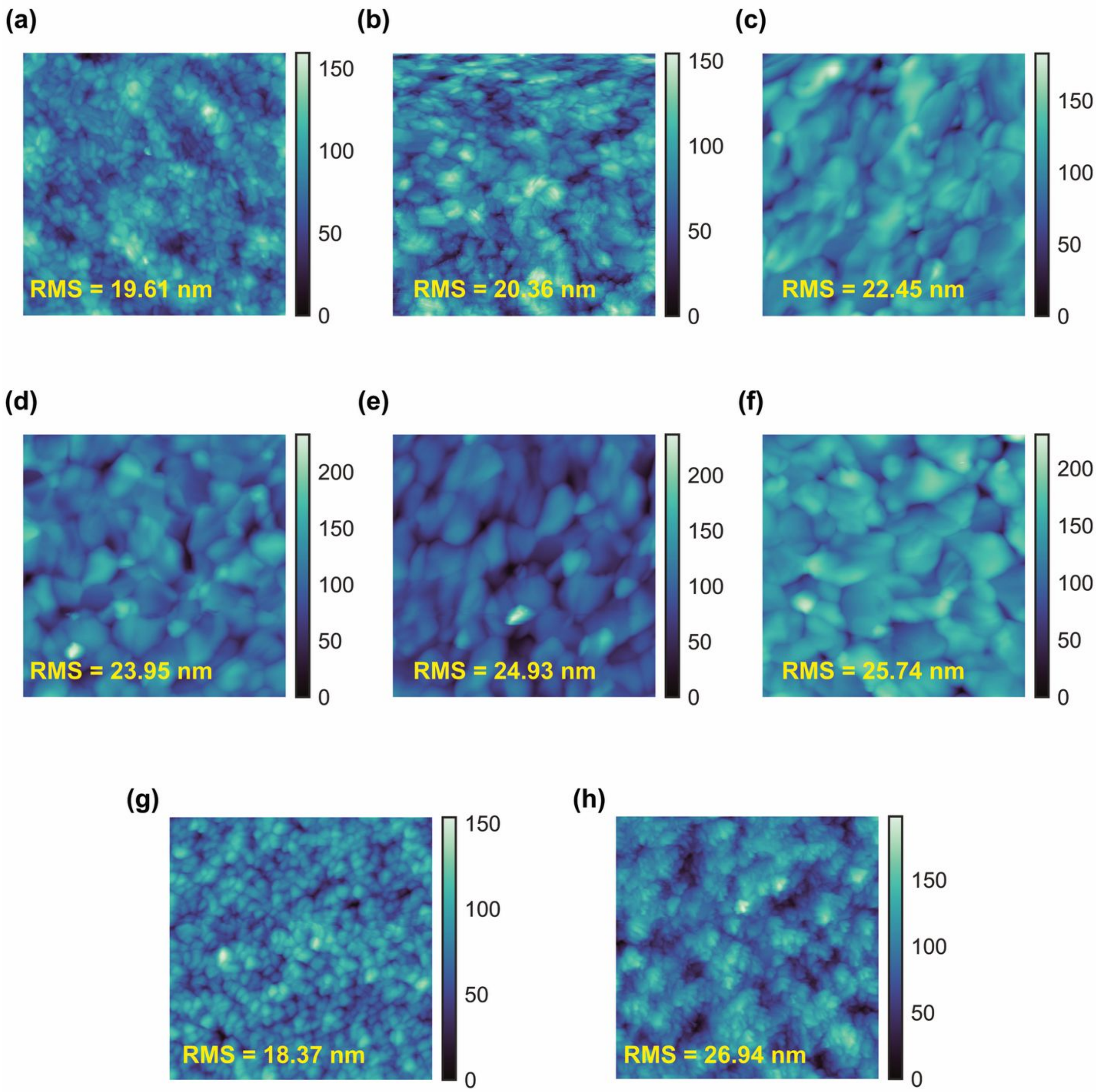


**Figure S11.** AFM images of (a) Control (b) MACl-10 (c) MACl-20 (d) MACl-30 (e) MACl-35 (f) MACl-40 (g) MCP-5 and (h) MCP-10 films.

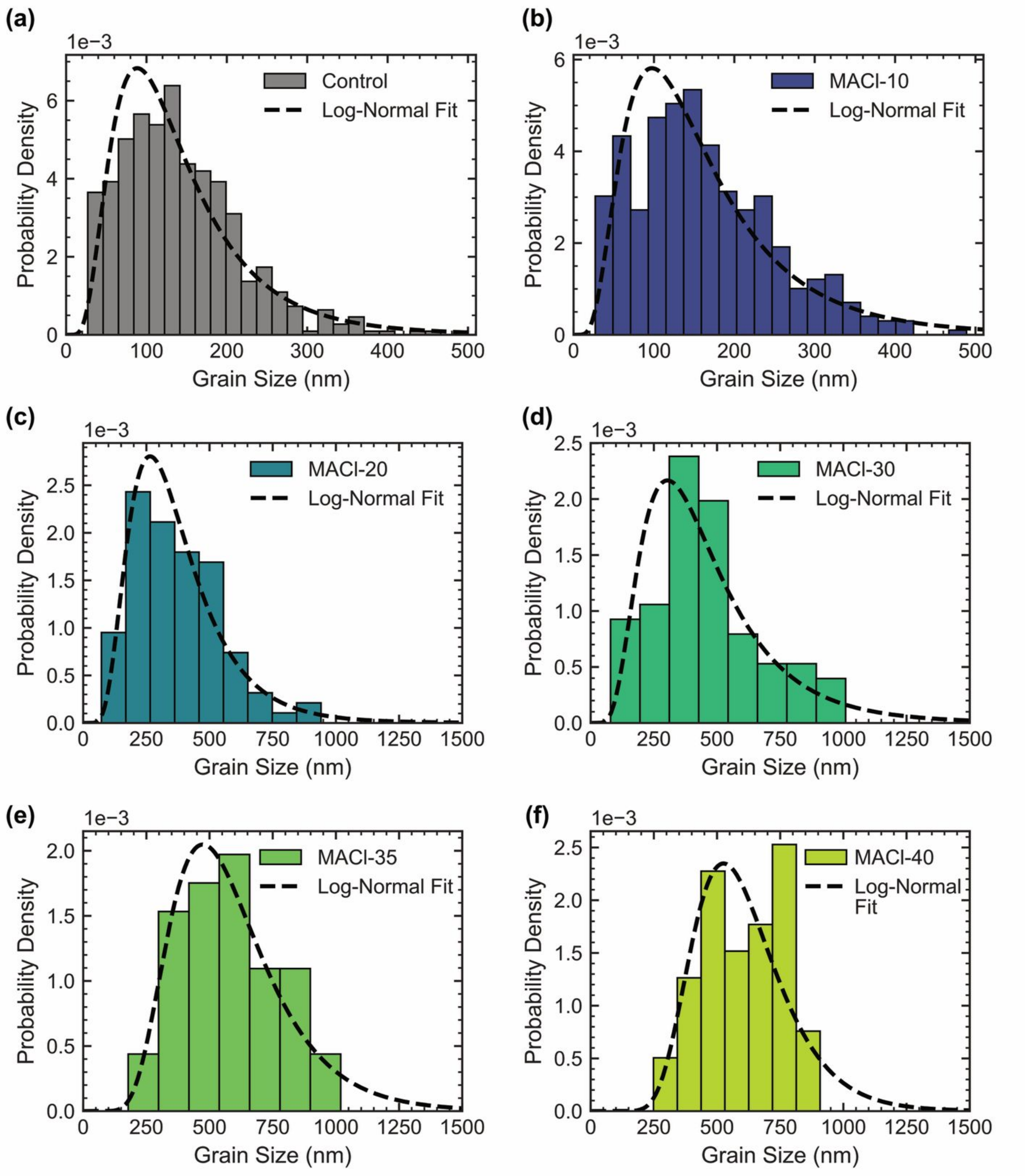
(a)
1e−3
Control
Log-Normal Fit
Probability Density
Grain Size (nm)
(b)
1e−3
MACl-10
Log-Normal Fit
Probability Density
Grain Size (nm)
(c)
1e−3
MACl-20
Log-Normal Fit
Probability Density
Grain Size (nm)
(d)
1e−3
MACl-30
Log-Normal Fit
Probability Density
Grain Size (nm)
(e)
1e−3
MACl-35
Log-Normal Fit
Probability Density
Grain Size (nm)
(f)
1e−3
MACl-40
Log-Normal Fit
Probability Density
Grain Size (nm)

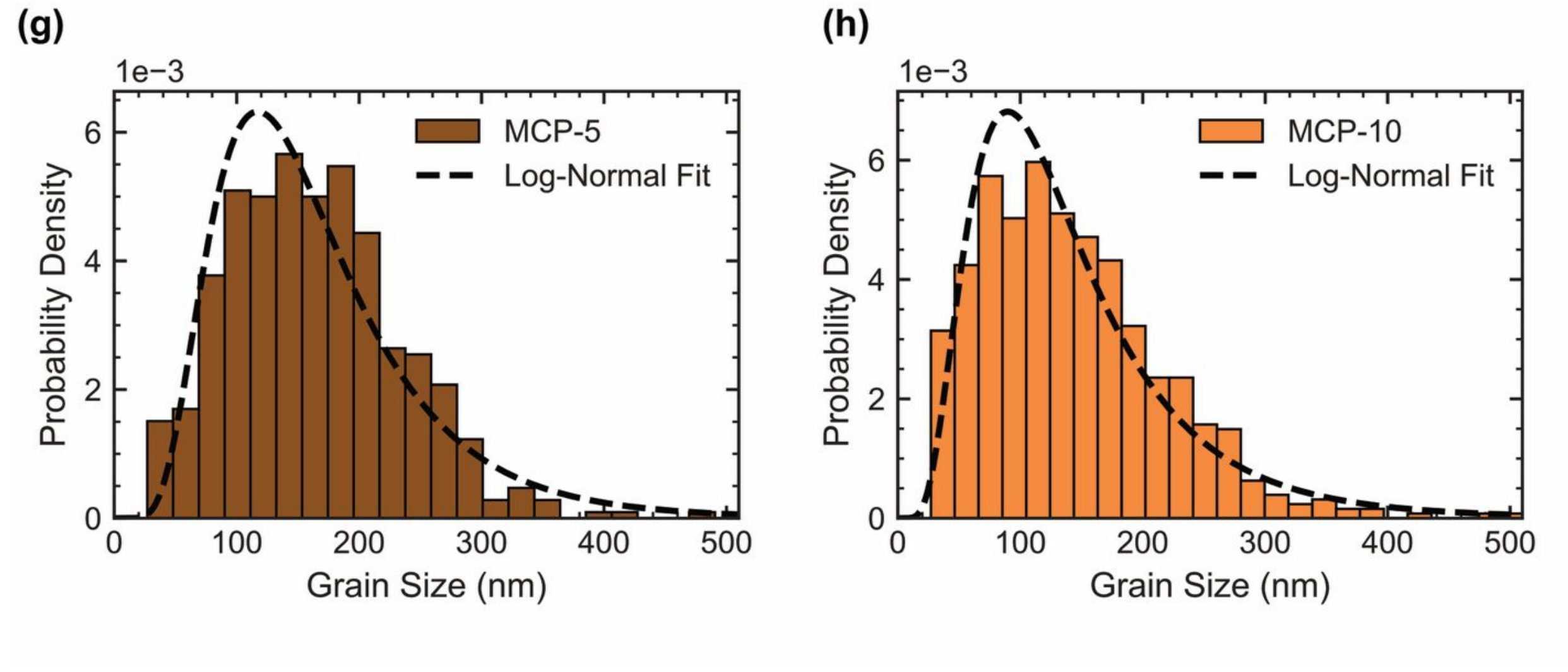


**Figure S12.** Grain size distribution of (a) Control, (b) MACl-10, (c) MACl-20, (d) MACl-30, (e) MACl-35, (f) MACl-40, (g) MCP-5, and (h) MCP-10 films

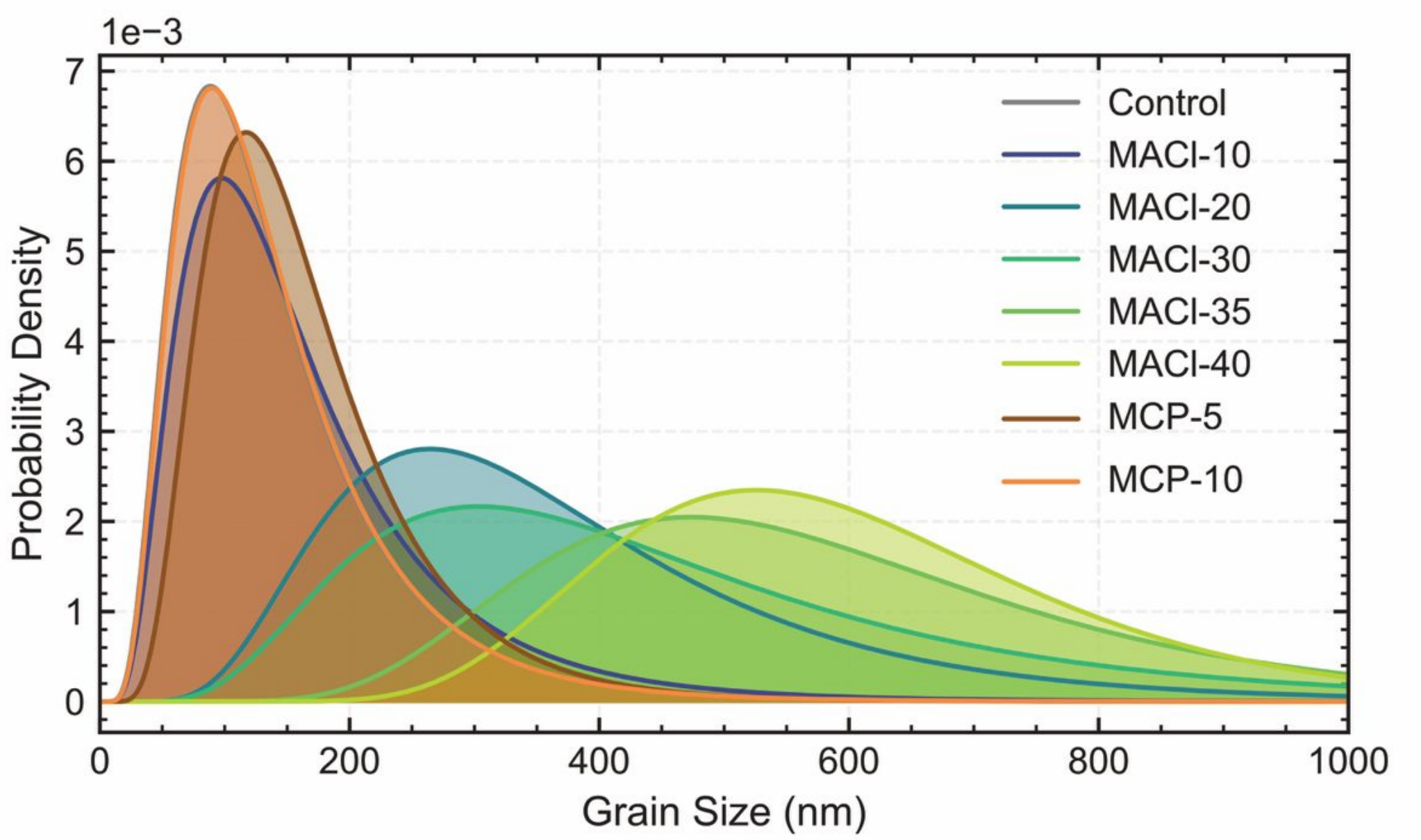


**Figure S13.** Comparing grain size distribution of MACl and MCP-5-treated samples with Control.

**Table S5.** Average grain size and standard deviation of perovskite films with and without MACl and MCP additives

| Sample | Mean Grain size | Standard deviation |
|---|---|---|
| **Control** | 142.3525629 | 86.96688946 |
| **MACl-10** | 164.6989863 | 106.462433 |
| **MACl-20** | 373.3655792 | 189.7543683 |
| **MACl-30** | 460.9599596 | 261.1236875 |
| **MACl-35** | 589.5290643 | 233.9589824 |
| **MACl-40** | 606.3093716 | 191.2792247 |
| **MCP-5** | 165.5285905 | 84.32055679 |
| **MCP-10** | 143.235919 | 86.67269362 |

# Power Spectral Density (PSD) Analysis

## Overview

Power spectral density (PSD) analysis was employed to quantify surface roughness and topographical characteristics from atomic force microscopy (AFM) images, providing a scale-dependent assessment of film morphology across multiple spatial frequencies. Unlike conventional statistical roughness metrics (e.g., RMS, Ra), PSD analysis captures both vertical and lateral components of surface structure, enabling comprehensive morphological comparison across all additive conditions.

### Mathematical Framework

The 2D isotropic PSD was calculated from AFM topographic data according to:

$$S(f) = \frac{1}{L^2 \Delta L^2} \left| \sum_{m=1}^{N} \sum_{n=1}^{N} Z_{mn} e^{-i2\pi(f_x m + f_y n)} \right|^2$$

Where:

- S(f) = 2D isotropic PSD at spatial frequency f ($nm^{-2}$)
- L = lateral scan dimension (µm)
- N = pixel resolution per direction (pixels)
- ΔL = sampling interval (L/N)
- $Z_{mn}$ = topographic height at pixel position (m, n)
- $F_x$, $f_y$ = spatial frequencies in x and y directions ($nm^{-1}$)

Polar averaging was then applied to reduce the 2D spectrum to a 1D radially averaged function:

$$S(f) = \frac{1}{2\pi}\int_0^{2\pi} S(f,\phi)\ d\phi$$

where φ is the azimuthal angle. This yields a frequency-dependent roughness representation independent of crystallographic orientation.

The k-correlation (ABC) Model: To quantify the surface parameters from the PSD profiles, the k-correlation model (also known as the ABC model) was applied. This model is particularly effective for simulation the low-frequency "knee" region of the spectrum, which marks the transition from frequency-independent to frequency-dependent roughness behavior. The model is expressed as:

$$PSD_{ABC}(f) = \frac{A}{(1 + B^2 f^2)^{\frac{C+1}{2}}}$$

Where A, B, and C are fitting coefficients:

- A: Spectral length at low frequencies
- B: Correlation length, representing the lateral scale of the surface features (e.g., grain size or feature spacing.
- C: Exponent related to the power-law fall-off at high frequencies.

Equivalent RMS roughness ($\sigma_{ABC}$) is derived from the k-correlation fitting parameters, providing a roughness value that accounts for the specific spectral distribution modeled by the fit. It's calculated as follows:

$$\sigma^2_{ABC} = \frac{2\pi A}{B^2(C-1)}$$

This approach allows for a more accurate comparison of surface texture by isolating the correlation length (B) and spectral strength (A) from the scanning scale effects often present in standard statistical analysis.

**Table S6**: k-correlation (ABC) model fitting parameters and calculated equivalent RMS roughness derived from Power Spectral Density (PSD) analysis of 5 x 5 $\mu m^2$ AFM images of perovskite films

| Sample | RMS (nm) | $RMS_{ABC}$ (nm) | $A_{ABC}$ (nm) | $B_{ABC}$ (nm) | $C_{ABC}$ |
|---|---|---|---|---|---|
| **Control** | 19.61 | 16.94 | 43256049 | 669.8667566 | 3.110970949 |
| **MACl-10** | 20.36 | 21.12 | 83677931.74 | 802.5250744 | 2.829729531 |
| **MACl-20** | 22.45 | 19.47 | 128699944.5 | 952.1261468 | 3.35358911 |
| **MACl-30** | 24.93 | 34.94 | 1028917863 | 1541.408256 | 3.229029114 |
| **MACl-35** | 23.95 | 34.4 | 1146837056 | 1629.361261 | 3.293173037 |
| **MACl-40** | 25.74 | 29.99 | 440377690.7 | 1153.478147 | 3.310720105 |
| **MCP-5** | 18.37 | 17.56 | 27375371.8 | 432.838209 | 3.976162467 |
| **MCP-10** | 26.94 | 26.06 | 189924655.7 | 939.4744674 | 2.989839463 |

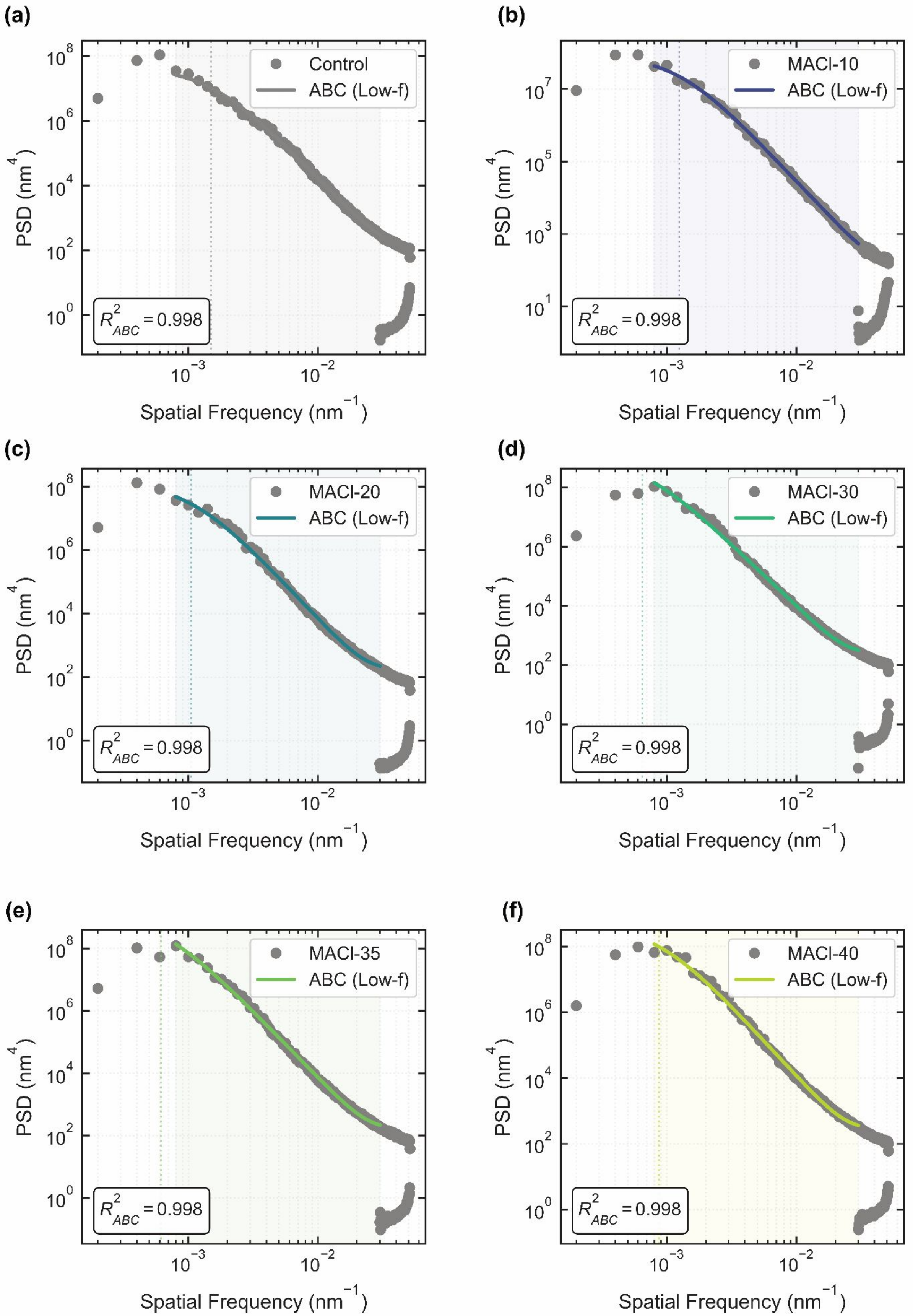

(a)
Control
ABC (Low-f)
$R^2_{ABC} = 0.998$
(b)
MACl-10
ABC (Low-f)
$R^2_{ABC} = 0.998$
(c)
MACl-20
ABC (Low-f)
$R^2_{ABC} = 0.998$
(d)
MACl-30
ABC (Low-f)
$R^2_{ABC} = 0.998$
(e)
MACl-35
ABC (Low-f)
$R^2_{ABC} = 0.998$
(f)
MACl-40
ABC (Low-f)
$R^2_{ABC} = 0.998$
PSD ($nm^4$)
Spatial Frequency ($nm^{-1}$)

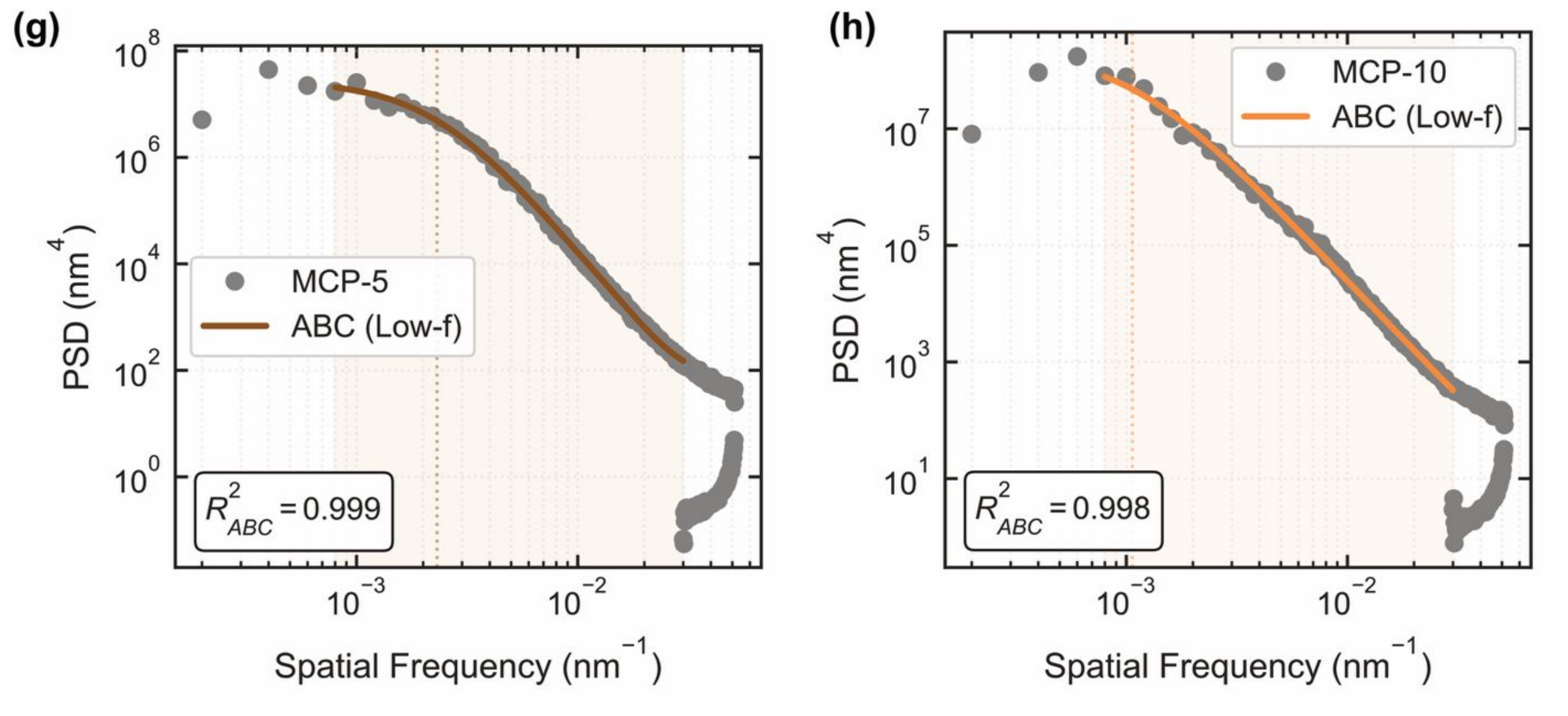


**Figure S14.** PSD profiles of (a) Control, (b) MACl-10, (c) MACl-20, (d) MACl-30, (e) MACl-35, (f) MACl-40, (g) MCP-5, and (h) MCP-10 films.

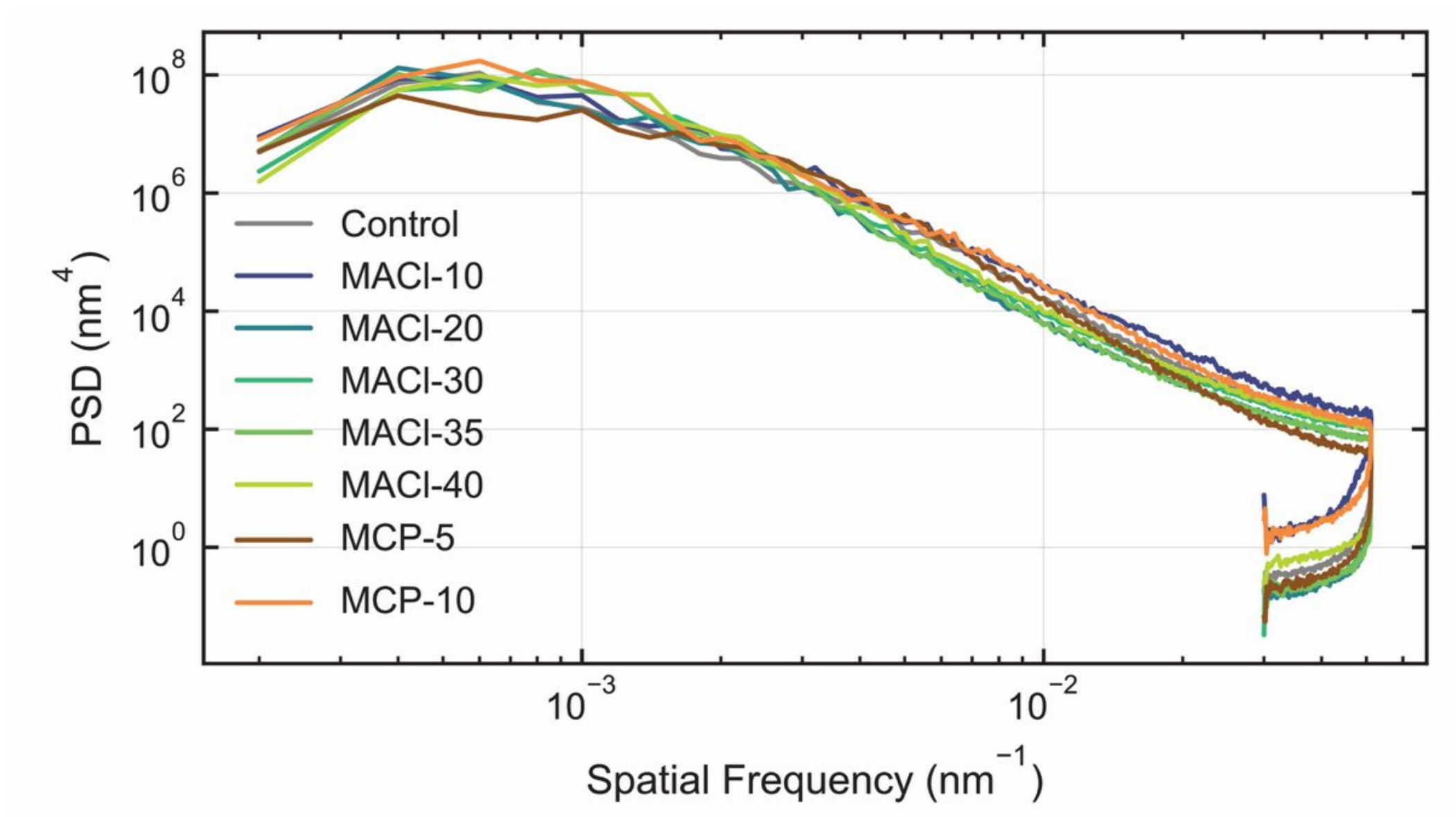


**Figure S15**. Comparison of the PSD profiles of all samples studied

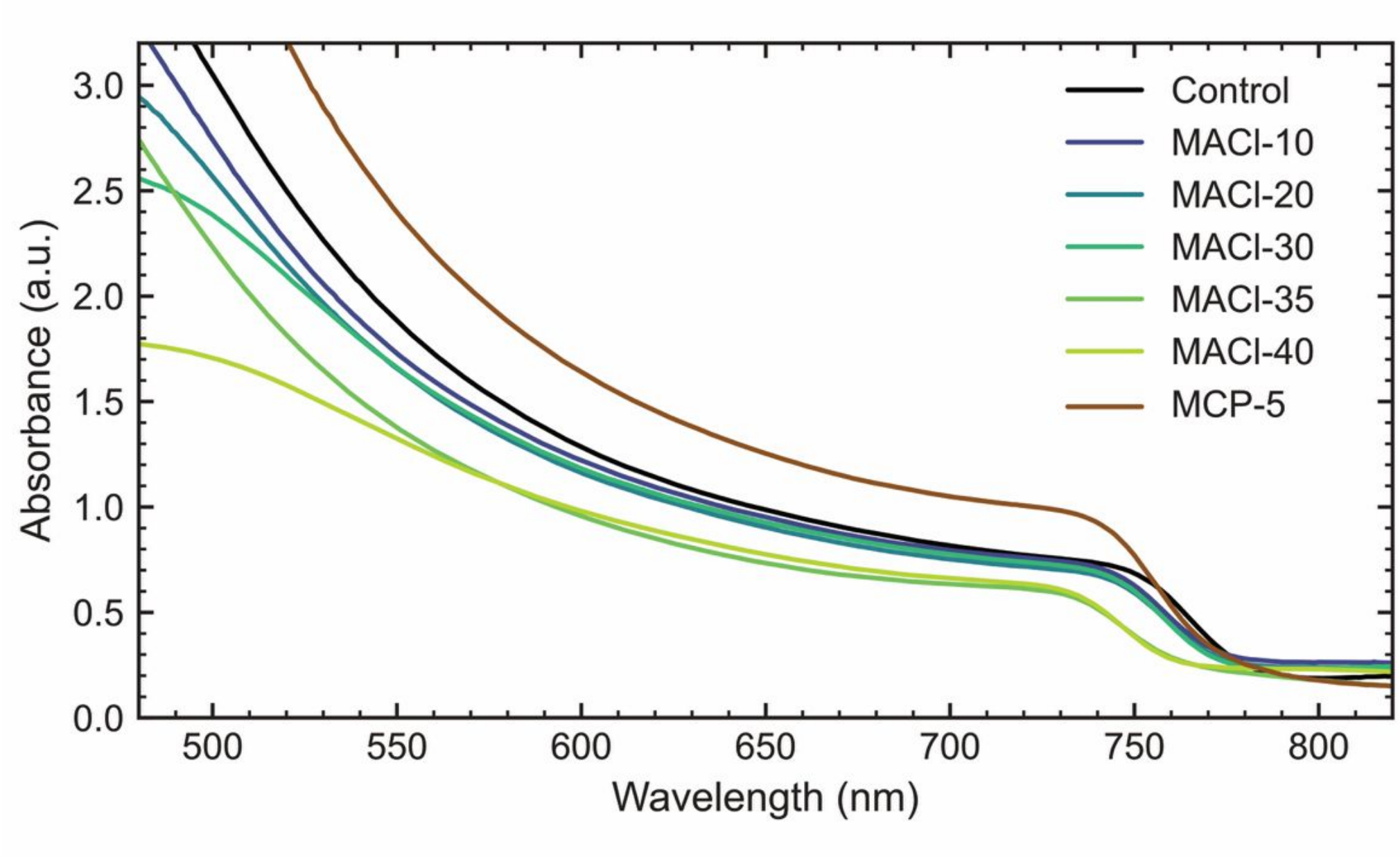


**Figure S16.** UV−Vis absorption spectra of all studied films from control to MACl-10 to MACl-40 and MCP-5 to MCP-10.

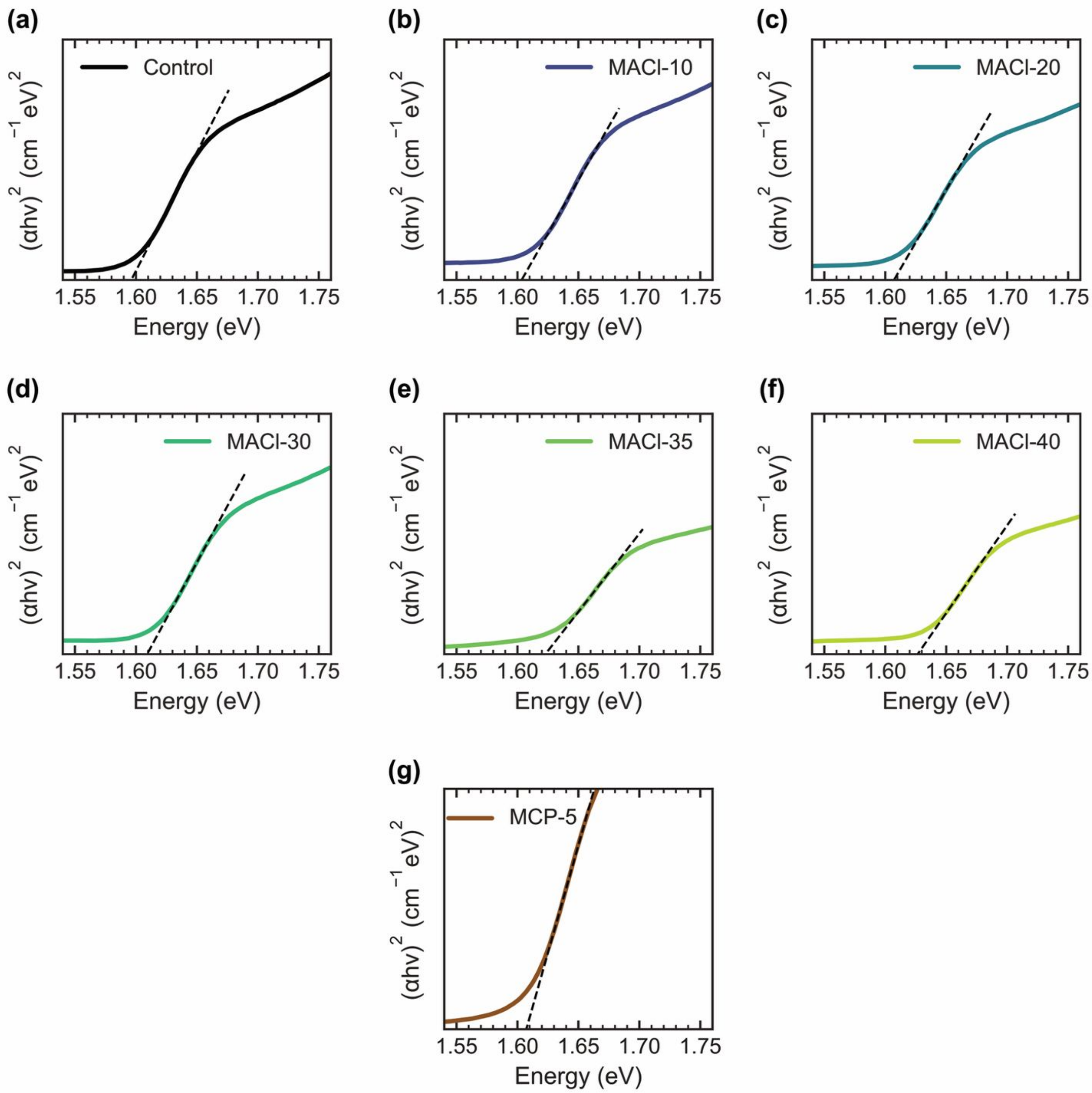


**Figure S17.** Tauc plot analysis of (a) Control, (b) MACl-10, (c) MACl-20, (d) MACl-30, (e) MACl-35, (f) MACl-40, and (g) MCP-5 films.

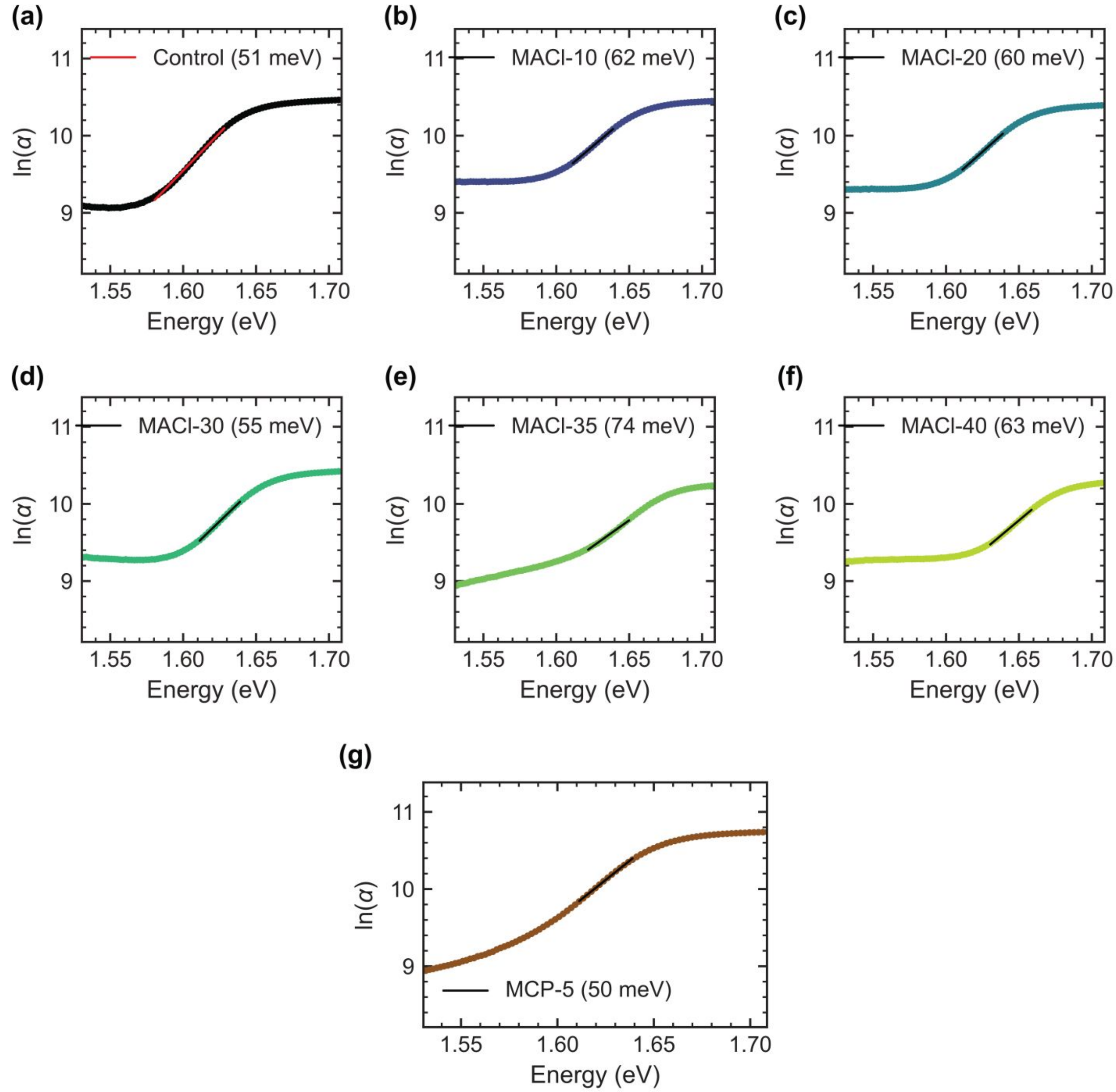


**Figure S18.** Urbach energy analysis of (a) Control, (b) MACl-10, (c) MACl-20, (d) MACl-30, (e) MACl-35, (f) MACl-40, and (g) MCP-5 films.

**Table S7.** Fitted TRPL parameters of perovskite films with and without the two chloride-based additives

| Sample | $A_1$ | $\tau_1$ (ns) | $A_2$ | $\tau_2$ (ns) | $\tau_{avg}$ (ns) |
|---|---|---|---|---|---|
| **Control** | 1023.82 | 7.14 | 214.27 | 210.49 | 182.14 |
| **MACl-20** | 259.064 | 113.5342 | 417.236 | 877.1278 | 820.32 |
| **MCP-5 (1)** | 331.712 | 442.5118 | 576.104 | 1487.3454 | 1334.54 |
| **MCP-5 (2)** | 349.242 | 43.0635 | 343.303 | 1431.6846 | 1390.46 |
| **MCP-5 (3)** | 353.277 | 27.7363 | 315.080 | 1562.9552 | 1533.00 |
| **MCP-5 (4)** | 318.843 | 59.1473 | 583.684 | 1693.6192 | 1663.02 |

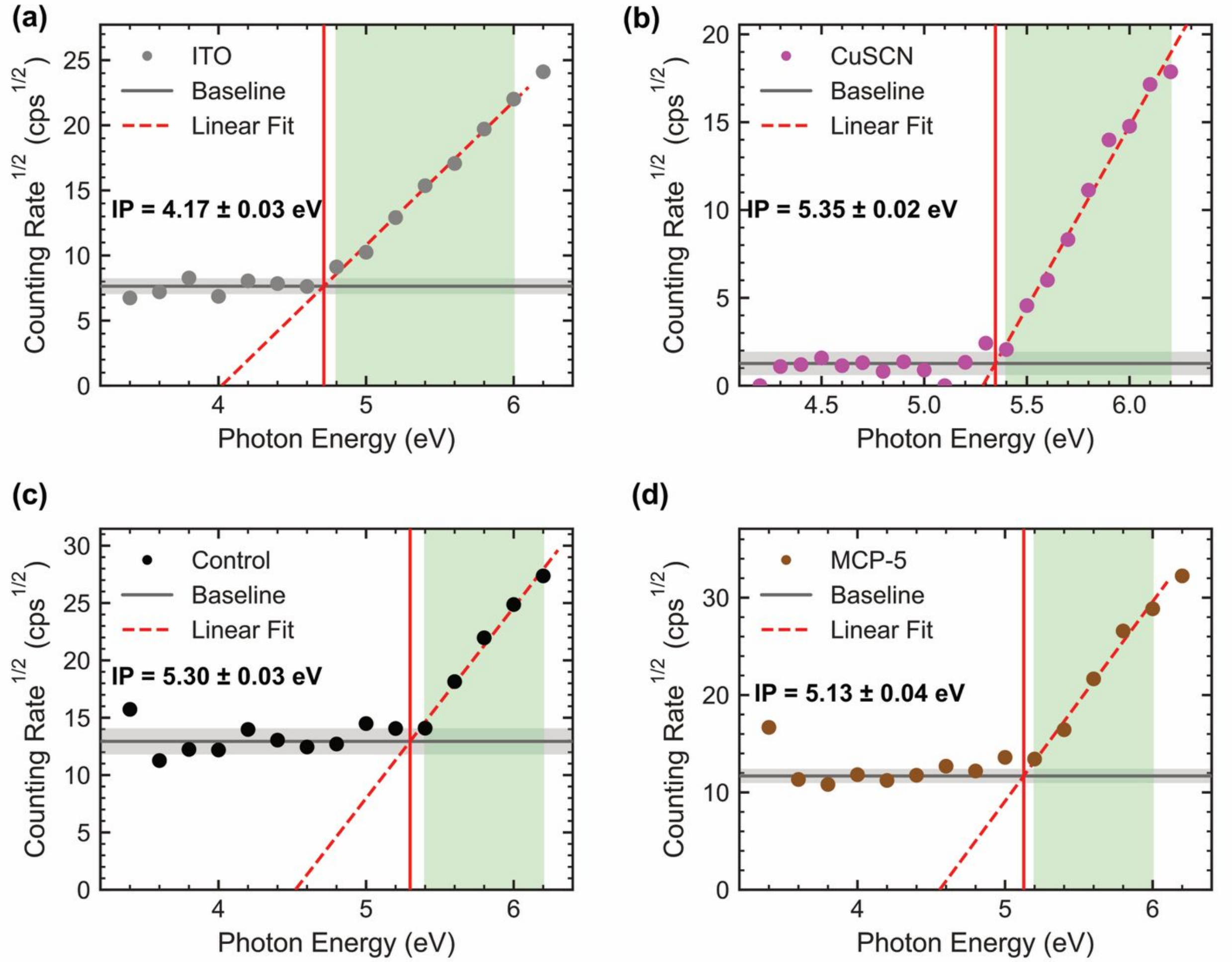


**Figure S19.** PYSA spectra of (a) control, (b) CuSCN HTL, (c) Control, and (d) MCP-5-treated film to determine the ionization potential (IP).

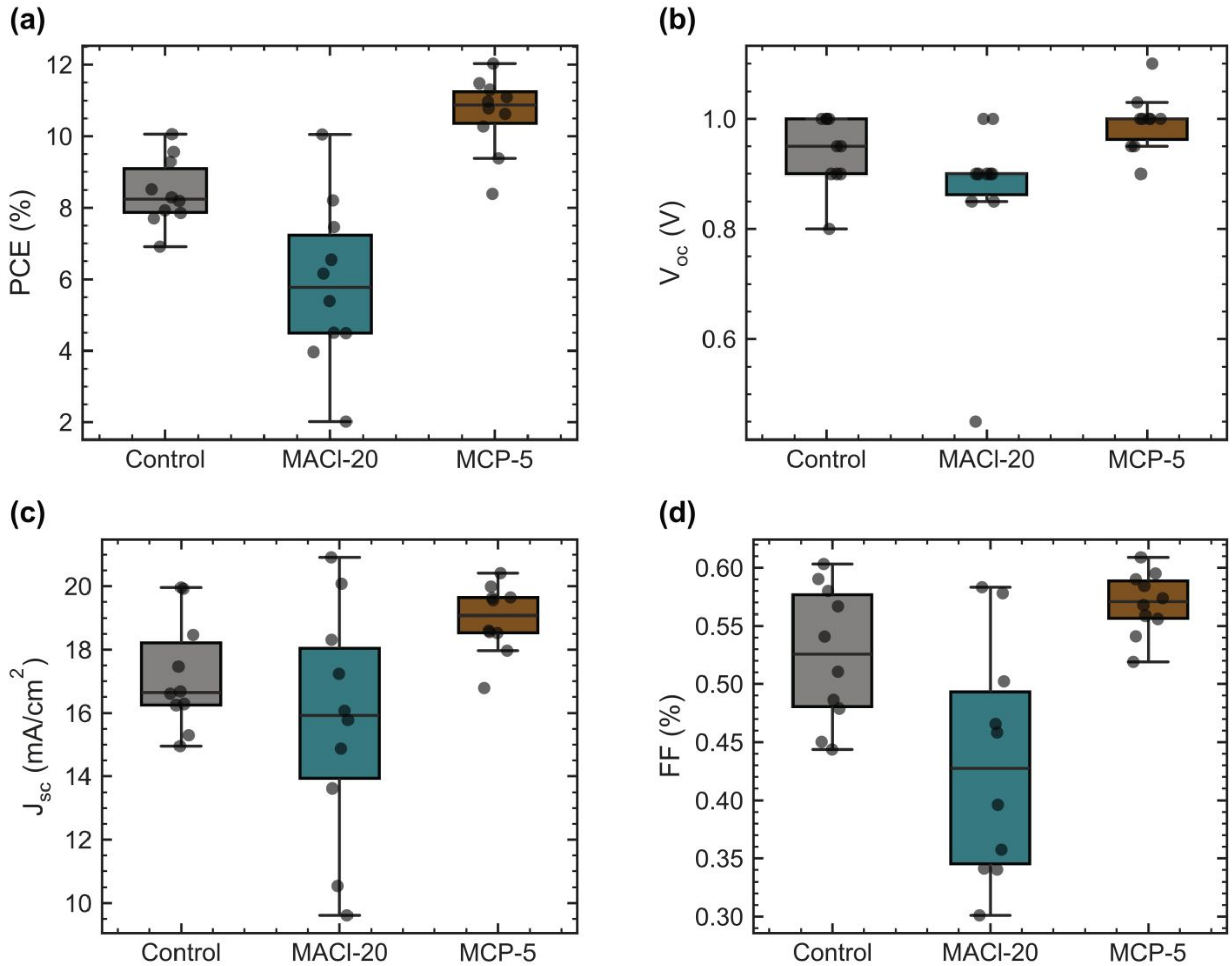


**Figure S20.** Statistical boxplot of photovoltaic parameters (a) PCE, (b) $V_{OC}$, (c) $J_{SC}$, and (d) FF of PSCs with and without the two chloride-based additives: control, MACl-20, and MCP-5
